# Interleukin-6 Gene Polymorphisms, Dietary Fat Intake, Obesity and Serum Lipid Concentrations in Black and White South African Women

**DOI:** 10.3390/nu6062436

**Published:** 2014-06-24

**Authors:** Yael T. Joffe, Lize van der Merwe, Juliet Evans, Malcolm Collins, Estelle V. Lambert, Alison September, Julia H. Goedecke

**Affiliations:** 1 UCT/MRC Research Unit for Exercise Science and Sports Medicine, Department of Human Biology, University of Cape Town, Cape Town 7505, South Africa; E-Mails: yael.joffe@gmail.com (Y.T.J.); lize@lizestats.co.za (L.M.); juliet.evans@westerncape.gov.za (J.E.); malcolm.collins@uct.ac.za (M.C.); vicki.lambert@uct.ac.za (E.V.L.); alison.september@uct.ac.za (A.S.); 2 Department of Statistics, University of Western Cape, Cape Town 7505, South Africa; 3 Non-communicable Disease Research Unit (NCDRU), South African Medical Research Council, PO Box 19070, Tygerberg, Cape Town 7505, South Africa

**Keywords:** *IL-6* gene, dietary fat, obesity, serum lipids, ethnicity

## Abstract

This study investigated interactions between dietary fat intake and *IL-6* polymorphisms on obesity and serum lipids in black and white South African (SA) women. Normal-weight and obese, black and white women underwent measurements of body composition, serum lipids and dietary fat intake, and were genotyped for the *IL-6* −174 G>C, IVS3 +281 G>T and IVS4 +869 A>G polymorphisms. In black women the IVS4 +869 G allele was associated with greater adiposity, and with increasing dietary fat intake adiposity increased in the IVS3 +281 GT+GG and IVS4 +869 AA or AG genotypes. In white women, with increasing omega-3 (*n*-3) intake and decreasing *n*-6:*n*-3 ratio, body mass index (BMI) decreased in those with the −174 C allele, IVS3 +281 T allele and IVS4 +869 AG genotype. In the white women, those with the IVS3 +281 T allele had lower triglycerides. Further, with increasing *n*-3 polyunsaturated fatty acid (PUFA); triglyceride and total cholesterol:high-density lipoprotein cholesterol (T-C:HDL-C) ratio decreased in those with the −174 C allele. In black women, with increasing total fat intake, triglycerides and T-C:HDL-C ratio increased in those with the *IVS4* +869 G allele. This study is the first to show that dietary fat intake modulates the relationship between the *IL-6* −174 G>C, *IVS3* +281 G>T and *IVS4* +869 A>G polymorphisms on obesity and serum lipids in black and white SA women.

## 1. Introduction

The cytokine interleukin 6 (IL-6) is known to regulate inflammation [1]. Higher circulating concentrations of IL-6 have also been associated with obesity and visceral adipose tissue (VAT) deposition [2,3,4], lipid metabolism [1] and increased risk for cardiovascular disease (CVD) [5]. In addition, there is a growing body of evidence linking polymorphisms within the *IL-6* gene to increased risk of obesity and dyslipidaemia [1,6,7,8,9]. Within the *IL-6* gene, the most frequently studied polymorphism is the *IL-6* −174 G>C polymorphism (rs1800795). This polymorphism is functional, with most studies showing the C allele to be associated with raised IL-6 and the acute phase protein, C-reactive protein (CRP) concentrations [5,10,11,12,13]. However, association studies between this *IL-6* gene polymorphism, obesity and dyslipidaemia have yielded conflicting results. A recent large meta-analysis by Yu *et al.* [10,14] found the −174 G>C polymorphism to be associated with obesity [13] which was not repeated in two other meta-analyses. Although Qi *et al.* [10] found no association between the −174 G>C polymorphism and obesity, they identified an *IL-6* haplotype to be associated with adiposity in healthy American men and women. Several studies have also found an association between the *IL-6* −174 G>C polymorphism and serum lipid concentrations, independent of obesity, with most showing the −174 G allele to be associated with higher total cholesterol (T-C), low-density lipoprotein cholesterol (LDL-C) and triglyceride (TAG) concentrations [6,8,15], and lower high-density lipoprotein cholesterol (HDL-C) concentrations [8,16].

Differences in study findings may relate to differences in environmental factors, such as dietary intake. Indeed, several studies have shown that dietary fat intake modulates the relationship between the inflammatory gene tumor necrosis alpha (*TNFA*) and obesity risk and dyslipidaemia [17,18,19,20,21,22]. However, to our knowledge, only two studies have reported on the relationship between any *IL-6* polymorphisms and dietary intake, and both studies investigated the *IL-6* −174 G>C polymorphism. Corpeleijn *et al.* [23] reported that the ability to increase fat oxidation after a high fat load was increased in obese European Caucasians with the *IL-6* −174 C allele. In Spanish men and women with a high CVD risk, the −174 CC genotype was associated with higher levels of adiposity at baseline, however after three years of nutritional intervention, those with the −174 CC genotype following a Mediterranean-style diet, had the greatest reduction in body weight [24].

Taken together, these findings suggest that this polymorphism is a strong candidate for obesity and dyslipidaemia. However, the −174 G>C polymorphism is rare in persons of African descent [25]. Therefore, the aims of this study were to: (i) identify informative (more common) polymorphisms in both black and white SA populations; (ii) investigate associations between these *IL-6* polymorphisms and obesity and dyslipidaemia; and (iii) explore interactions between these *IL-6* polymorphisms and dietary fat intake on obesity and serum lipids in black and white South African (SA) women.

## 2. Experimental Section

### 2.1. Subjects

A convenience sample of 107 normal-weight (body mass index (BMI)) < 25 kg/m^2^) and 120 obese (BMI ≥ 30 kg/m^2^) black and 89 normal-weight and 62 obese white SA women between the ages of 18 and 45 years were recruited in the greater Cape Town area, from 2004 to 2006, as described previously. Inclusion criteria for this study included: no previous diagnosis or undergoing therapy for diabetes, hypertension, HIV or other metabolic diseases, not currently pregnant or lactating. For diet-gene interactions, only 73 normal-weight and 74 obese black, and 73 normal-weight and 48 obese white adequate reporters were included in the analysis.

Approval was obtained from the Human Research Ethics Committee of the Faculty of Health Sciences at the University of Cape Town, and written informed consent was obtained from all participants. The study was performed in accordance with the principles of the Declaration of Helsinki, ICH Good Clinical Practice.

### 2.2. Measurements

#### 2.2.1. Body Composition

Basic anthropometric measurements including weight, height, and waist and hip circumferences were taken, and whole body composition was measured using dual-energy X-ray absorptiometry (Hologic Discovery-W, software version 12.1, Hologic Inc., Bedford, MA, USA).

#### 2.2.2. Blood Sampling and Analysis

Blood samples were drawn from the antecubital vein after an overnight (10–12 h) fast for the determination of serum TAG, T-C, HDL-C, and LDL-C concentrations, and for DNA extraction. Serum TAG, T-C, and HDL-C concentrations were measured on the Roche Modular Auto Analyzer using enzymatic colorimetric assays. LDL-C was calculated using the Friedewald equation [26].

#### 2.2.3. Polymorphism Selection

The *IL-6* gene spans a physical region of 4.8 kb on chromosome seven and includes five exons and four introns. The most commonly studied *IL-6* polymorphism is the G>C functional polymorphism at position −174 in the promoter region of the gene. The frequency of this polymorphism was expected to be very low (<5%) in the black SA population [25,27]. Therefore, in addition to the *IL-6* −174 G>C polymorphism, additional polymorphisms with a reported high heterozygosity in both the white and black SA populations were identified. Only two such polymorphisms were identified in both the black and white populations and both localise to the non-coding regions of the *IL-6* gene; rs1554606: *IVS3* +281 G>T (intron 3) and rs2069845: *IVS4* +869 A>G (intron 4). These polymorphisms altered restriction enzyme recognition sequences, facilitating restriction fragment length polymorphism analysis (RFLP) and were therefore considered suitable for this study.

#### 2.2.4. DNA Extraction and Genotype Analysis

DNA was extracted using the method of Lahiri and Nurnberger [28]. Genotyping of the −174 G>C polymorphism (rs1800795) within the promoter of the *IL-6* gene was performed by polymerase chain reaction (PCR) amplification and digested with the *SfaN1* restriction enzyme as previously described [15]. Several control samples (no DNA) were included on each 96 well PCR plate together with several repeat DNA samples as a measure to detect PCR contamination and potential genotyping errors. In addition, several samples were randomly selected and these were repeated to confirm genotype scores. All these controls and random samples were repeatedly scored the same on all plates. This was done for all polymorphisms genotyped.

#### 2.2.5. *IL-6* IVS3 +281 G>T, rs1554606

Standard PCR amplification reactions were conducted using forward primer 5′-GTACCAACTTGTCGCACTCA-3′ and reverse primer 5′-GGATCCTTCTCTGATTGTCC-3′. PCR was conducted using a thermal cycler (Hybaid; PCR Express, Middlesex, UK) under the following conditions: 5 min at 94 °C, 30 cycles of 30 s at 94 °C, 30 s at 58 °C, and 40 s at 72 °C, followed by a final extension step for 5 min at 72 °C. The 472 bp PCR product was digested with *Dde I* producing fragments of 34 bp, 191 bp, and 247 bp for the T allele and 34 bp, and 438 bp for the G allele.

#### 2.2.6. *IL-6* IVS4 +869 A>G, rs2069845

Standard PCR amplification reactions were conducted using the forward primer 5′-GAGTCTGACTTAGCAAGCCTCCGGT-3′ with sites created for *Msp1* and the reverse primer 5′-CCAAGCCTGACCAGCATCACTATC-3′. PCR was conducted using a thermal cycler (Hybaid; PCR Express, Middlesex, UK) under the following conditions: 5 min at 94 °C followed by 30 cycles of 30 s at 94 °C, 30 s at 58 °C, and 40 s at 72 °C, followed by a final extension step for 5 min at 72 °C. The 383 bp PCR product was digested with *Msp1* producing fragments of 21 bp, 155 bp, and 207 bp for the G allele and 21 bp and 362 bp for the A allele. All resultant fragments were resolved on 6% polyacrylamide gels and visualised by SYBER^®^ Gold staining.

#### 2.2.7. Dietary Assessment

Dietary intake was estimated using a validated quantified food frequency questionnaire (QFFQ) [29,30]. Nutrient intake was calculated by means of the software program FoodFinder, III™ (South African Medical Research Council, Cape Town, South Africa) [31]. Under-reporting and over-reporting were detected on the basis of the ratio of energy intake (kJ/day): estimated resting metabolic rate (kJ/day, Harris Benedict equation [32,33]). Upper and lower cut-points were calculated assuming a sedentary lifestyle of 1.55× basal metabolic rate as defined by the physical activity level values from the World Health Organization (WHO) recommended energy requirements [34]. Participants were only included in the diet-gene analysis if this ratio was between 1.05 and 2.28 [32,33]. After applying these cut-off points, only 74 white and 73 black normal-weight, and 48 white and 73 black obese adequate reporters were included in all analyses. There were no differences between the over- and under-reporters, and the adequate-reporters for body composition or metabolic outcomes (results not shown).

### 2.3. Statistical Analysis

In order to explore the metabolic effects of extreme ranges in BMI and hence most other measures of obesity and fat distribution, while maintaining a unimodal distribution for lipid profile, we included only normal-weight and obese women in the analyses. The obese women were older than the normal-weight women, and therefore all analyses were age-adjusted. Genotype and allelic frequency distributions were tested for association with BMI groups and ethnicity using logistic regression (a generalized linear model with binomial family and logit link), adjusting for each other. Exact tests of Hardy Weinberg Equilibrium (HWE) were used for the three *IL-6* polymorphisms in black and white women.

General linear models were used to model dietary intake, body composition, and lipid variables (outcomes) in black and white women, separately, and together in the same model, while adjusting for ethnicity. Outcomes were either left untransformed, or transformed (adding a constant, then taking the natural log), when required, to approximate normality, for analysis. For descriptive purposes, outcomes were modelled as functions of BMI group and the *IL-6* polymorphisms and their interactions. Interactions between dietary intakes and *IL-6* polymorphisms on outcomes, were tested by including interaction terms in the models. The models were adjusted for specific factors, by including them in the models. Tests of the above interaction on body composition variables were adjusted for age, whereas the interaction on lipid variables was adjusted for age and fat mass. Interactions were tested in white and black, separately.

When we found significant interactions, we presented graphs to aid in their interpretation, because interaction effects are difficult to describe. We illustrated six of the significant interactions between *IL-6* genotype and dietary fatty acid intake on fat mass and BMI respectively, by plotting the observed raw values as well as the modeled relationships for a woman 27.5 years old. The natural logs of the dietary intakes were used in the models, with the result that the modeled relationships of the untransformed values were curves, not straight lines. The curves will be slightly higher for older, but lower for younger women.

In order to determine whether interaction effects differed between ethnic groups, three-way interactions between ethnic group and dietary intakes and *IL-6* polymorphisms were modeled in the combined group (of white and black women). In order to determine whether interaction effects were the same in the two ethnic groups, an indicator of ethnic group was included (and tested) in models of the two-way interaction between dietary intakes and *IL-6* polymorphisms on outcome in the combined group.

Results corresponding to *p*-values below 5% are described as significant. We did not adjust for multiple testing because it has been suggested that corrections, such as Bonferroni, are too conservative when several associations are tested in the same group of individuals [35], and Nyhold is not appropriate in a situation such as this, where there are only three polymorphisms [35,36,37]. In addition, the association of the linked *IL-6* polymorphisms with body composition and serum lipids was based on an *a priori* hypothesis.

For the diet-gene interactions, a significant interaction suggests that there is a significant difference in rates of change between the groups. There are two possibilities: either only the difference is significant, but none of the individual rates of change are, or one or both of the “slopes” change significantly. In the results only the significant effects are described.

In this study, with 63 obese and 89 normal-weight white women and a minor allele frequency of 0.30 (observed for the *IVS4* +869 G allele), we have 81% power to detect an obesity odds ratio of 2. In addition, in this study, with 108 obese and 124 normal-weight black women and a minor allele frequency of 0.26 (observed for the *IVS4* +869 G allele), we have 93% power to detect an obesity odds ratio of 2.

Analyses were done in R, a language and environment for graphics and statistical computing [38,39]. The R package genetics was used for allele and genotype frequencies, HWE, and linkage disequilibrium (LD) probability testing.

## 3. Results

### 3.1. Subject Characteristics

Basic subject characteristics of the black and white women are summarised, by BMI group in Table 1. The white women were older than the black women; as a result all analyses were adjusted for age. The white women, within both BMI groups, had greater weight, waist, waist:hip ratio (WHR), than the black women, and the black women had higher BMI and body fat % than the white women. In addition, all serum lipid concentrations were higher in the white compared to the black women.

Irrespective of ethnicity, the obese women were older than the normal-weight women, and by design of the study, all body composition variables were higher in the obese compared to the normal-weight women. In addition, TAG, T-C, and LDL-C and were all significantly higher and serum HDL-C concentrations were lower in the obese compared to the normal-weight women (Table 1).

### 3.2. Genotype and Allelic Frequency

The genotype and minor allele frequency distribution of the *IL-6* polymorphisms investigated in this study for normal-weight and obese, black and white women are presented in Table 2. There were no significant differences between the normal-weight and obese women, after adjusting for ethnicity, for the *IL-6* −174 G>C genotype (*p* = 0.104) or allele frequencies (*p* = 0.515), *IL-6* IVS3 +281 G>T genotype (*p* = 0.914) or allele frequencies (*p* = 0.950), and *IL-6* IVS4 +869 A>G genotype (*p* = 0.293) or allele frequencies (*p* = 0.474).

However, the difference in the *IL-6* −174 G>C and IVS3 +281 G>T genotype distribution and allele frequency between black and white women was highly significant after adjusting for BMI group (*p* < 0.001). Although the IVS4 +869 A>G genotype distribution between black and white women was also significant after adjusting for BMI group (*p* < 0.001), the allele frequency was not different (*p* = 0.688). The frequency of the minor allele of the −174 G>C and IVS3 +281 G>T polymorphisms were higher in the white compared to the black women. All genotype frequencies reported in this study were similar to European and African populations reported in the Ensembl database, however none of the African populations reported in the Ensembl database were representative of populations from Southern Africa.

**Table 1:** Subject characteristics, body composition and serum lipids of normal-weight and obese black and white women.

Subject characteristics	Black, *n* = 147		White, *n* = 121		*p*-values		
	Normal-weight	Obese	Normal-weight	Obese	BMI Group	Ethnicity	Interaction
n	73	74	73	48	-	-	-
Age, years	24 ± 6	30 ± 8	28 ± 7	34 ± 9	<0.001	<0.001	0.969
*Body Composition* ^a^							
Height, m	1.61 ± 0.06	1.59 ± 0.06	1.67 ± 0.07	1.67 ± 0.07	0.009	<0.001	0.350
Weight, kg	58 ± 6	91 ± 13	61 ± 6	97 ± 14	<0.001	0.001	0.272
BMI, kg/m^2^	22 ± 2	36 ± 5	21 ± 2	35 ± 4	<0.001	0.005	0.596
Fat Mass, kg	17 ± 4	41 ± 9	17 ± 4	44 ± 9	<0.001	0.190	0.045
Body fat, %	31 ± 5	45 ± 4	28 ± 5	45 ± 4	<0.001	0.006	0.004
Waist, cm	74 ± 6	103 ± 12	77 ± 6	106 ± 10	<0.001	0.004	0.972
WHR	0.75 ± 0.06	0.83 ± 0.08	0.78 ± 0.05	0.85 ± 0.06	<0.001	<0.001	0.426
*Metabolic Outcomes* ^b^							
TAG, mmol/L	0.50 (0.40–0.70)	0.80 (0.60–1.04)	0.80 (0.60–1.08)	1.10 (0.78–1.50)	<0.001	<0.001	0.339
T-C, mmol/L	3.8 (3.3–4.2)	4.1 (3.6–4.5)	4.5 (4.0–5.0)	5.0 (4.4–5.7)	0.031	<0.001	0.357
HDL-C, mmol/L	1.5 (1.2–1.7)	1.1 (1.0–1.3)	1.8 (1.6–2.0)	1.5 (1.3–1.6)	<0.001	< 0.001	0.638
LDL-C, mmol/L	2.1 (1.6–2.5)	2.5 (2.0–2.9)	2.3 (1.9–2.7)	2.9 (2.6–3.5)	<0.001	< 0.001	0.215
T-C:HDL–C ratio	2.6 (2.2–3.1)	3.5 (2.9–4.1)	2.6 (2.2–2.8)	3.5 (3.0–4.2)	<0.001	0.520	0.260

TAG, triglycerides; T-C, total cholesterol; HDL-C, high density lipoprotein cholesterol; LDL-C, low density lipoprotein cholesterol; T-C:HDL-C ratio, total cholesterol: high density lipoprotein cholesterol ratio; %E, percentage of total energy intake; ^a^ Body composition:Summarised as mean and SD. *p*-values are from age adjusted linear models of the interaction between BMI group and genotype on variable. *p*-values were calculated consecutively, from left to right, each adjusted for age and factors already tested; ^b^ Lipids: Summarised as median (interquartile range). Outcomes were log-transformed for modelling. *p*-values are from age adjusted linear models of the interaction between BMI group and genotype on variable. *p*-values were calculated consecutively, from left to right, each adjusted for age and factors already tested.

**Figure 1:**
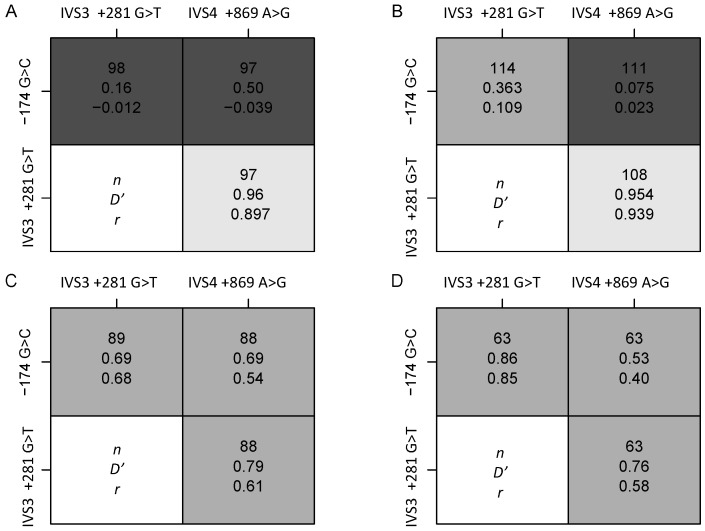
LD structure of *IL-6* polymorphisms (*n*, number of women; *Dʼ*, Lewontin’s *D*; *r*, coefficient of correlation) for *IL-6* polymorphisms of normal-weight and obese black and white women. LD plots showing (**A**): Normal Black; (**B**) Obese Black; (**C**) Normal White; (**D**) Obese White. Shading determined by value of *Dʼ*–light indicates high value.

Deviations from HWE were detected for the −174 G>C polymorphism in the black obese group and for the IVS4 +869 A>G polymorphism in both the normal-weight and obese white women (Table 2). Only one individual was homozygous for the IVS4 +869 G allele, despite the frequency of the minor G allele being over 30% in the white SA women. In the white women, all the polymorphisms were in high LD in both the normal-weight and obese groups. In the black women, of the three pairs of polymorphisms genotyped, only IVS4 +869 A>G and IVS3 +281 G>T were in LD, in both the normal-weight and obese groups (Figure 1).

**Table 2:** Genotype and minor allele frequencies of normal-weight and obese black and white women.

Subject characteristics	Normal-weight		Obese		* *p*-values		Ensemble 1000 Genomes: Phase 1			
	Black	White	Black	White	* Ethnicity	** BMI Group	EUR	GBR	AFR	ASW
*IL-6* 174 G>C rs1800795										
*n*	72	74	72	48	-	-	-	-	-	-
G/G	0.97	0.28	0.97	0.25	-	-	0.36	0.39	0.95	0.78
G/C	0.03	0.61	0.03	0.50	-	-	0.44	0.42	0.05	0.21
C/C	0.00	0.11	0.00	0.25	<0.001	0.104	0.20	0.19	0.0	0.0
C	0.01	0.41	0.01	0.50	<0.001	0.515	0.41	0.40	0.02	0.11
*** HWE *p*-value	1.000	0.053	1.000	1.000	-	-	-	-	-	-
*IL-6* 281 G>T Dde I rs1554606										
*n*	66	74	69	48	-	-	-	-	-	-
G/G	0.59	0.35	0.59	0.25	-	-	0.34	0.36	0.48	0.39
AG/T	0.35	0.47	0.36	0.54	-	-	0.45	0.44	0.46	0.57
T/T	0.06	0.18	0.04	0.21	<0.001	0.914	0.20	0.19	0.05	0.03
T	0.23	0.41	0.22	0.48	<0.001	0.950	0.43	0.41	0.28	0.32
*** HWE *p*-value	0.740	0.814	1.000	0.772	-	-	-	-	-	-
*IL-6* 869 A>G Msp I rs2069845										
*n*	65	73	65	48	-	-	-	-	-	-
A/A	0.55	0.38	0.58	0.29	-	-	0.34	0.36	0.46	0.37
G/A	0.35	0.60	0.37	0.71	-	-	0.45	0.44	0.47	0.57
G/G	0.09	0.01	0.05	0.00	<0.001	0.293	0.20	0.19	0.05	0.04
G	0.27	0.32	0.23	0.35	0.688	0.474	0.43	0.41	0.29	0.33
*** HWE *p*-value	0.526	0.001	1.000	<0.001	-	-	-	-	-	-

*p*-values are from age-adjusted models testing association with * ethnic groups, adjusting for BMI group; and ** BMI groups, adjusting for ethnicity; and *** exact tests of Hardy-Weinberg Equilibrium. Population frequencies are from the Ensemble public database [40]. EUR, European; GBR, British in England and Scotland; AFR, African; ASW, Americans of African Ancestry in SW USA.

### 3.3. Body Composition and Genotype

**Figure 2:**
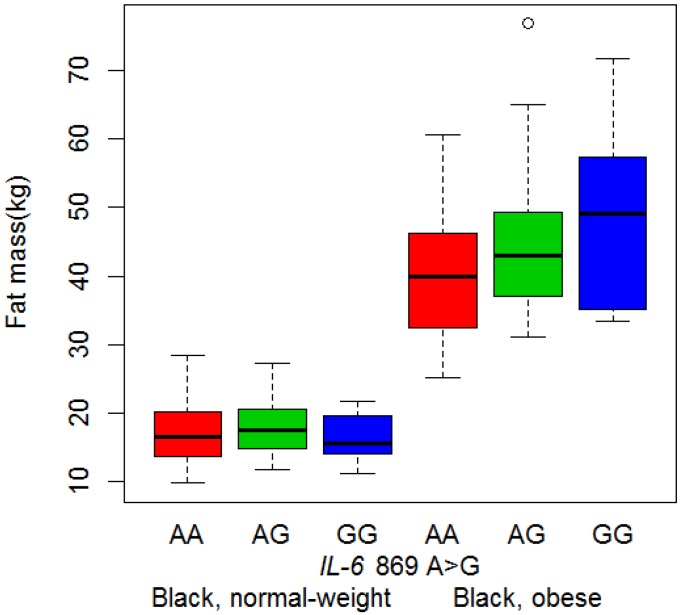
Interaction between *IL-6* IVS4 +869 A>G polymorphism and BMI group on fat mass in black women. Figure contains boxplots of fat mass in black women, separated by BMI group and genotype. Boxplots indicate the median, the quartiles and the minimum and maximum values for each group. Abbreviations: BMI, body mass index; *IL-6*, Interleukin-6.

The IVS4 +869 A>G was the only *IL-6* polymorphism to be associated with body composition. In the black women, waist circumference was 3.38 cm (95%CI: 0.79–5.98) higher in those with the AG and GG genotypes compared to the AA genotype (*p* = 0.011, dominant model). The association between this polymorphism and BMI group on fat mass in black women (*p* = 0.034) is illustrated in Figure 2. The differences in fat mass between genotypes in the normal-weight group were not significant, but in the obese group, each G allele added, on average, 4.28 kg (95%CI: 1.90–6.65) to fat mass (*p* = 0.010, additive allelic model).

The ethnic-specific nature of these genotype-phenotype associations were examined by including both black and white women in the same model and adjusting for ethnicity (Table S1). The associations between the IVS4 +869 A>G polymorphism and waist and fat mass described above were significant in the black women, but not in the white group, nor in the combined group (Table S1), suggesting that these associations were different for the black and white women.

### 3.4. Serum Lipid Concentrations and Genotype

**Figure 3:**
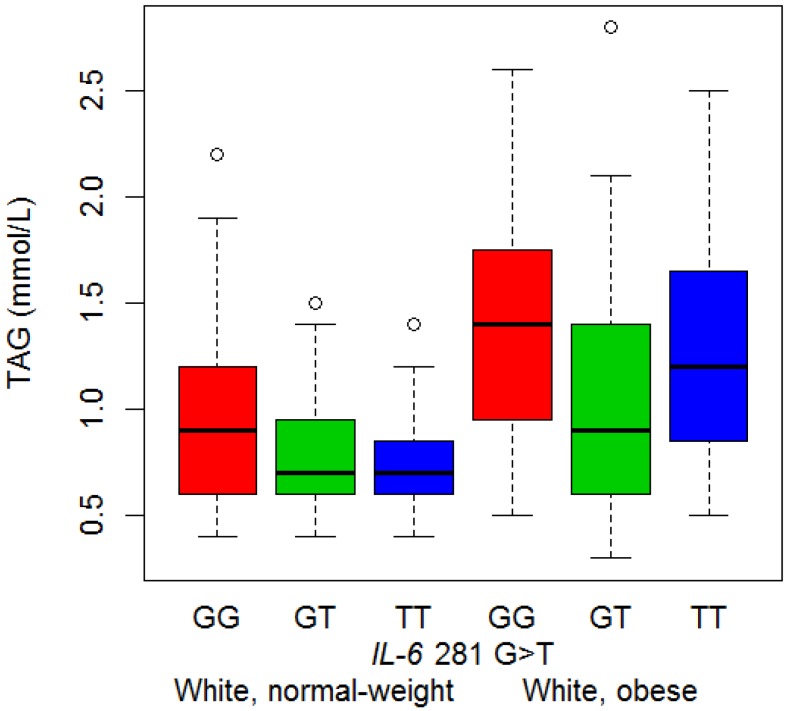
Association of *IL-6* IVS3 +281 G>T polymorphism with triglyceride concentration in white women. The figure contains boxplots of triglyceride concentrations in white women, separated by BMI group and *IL-6* IVS3 +281 G>T genotype.

The only association identified between the *IL-6* polymorphisms and serum lipid concentrations, was found in the white women, between the IVS3 +281 G>T polymorphism and serum TAG concentrations (*p* = 0.024) (Figure 3). White women with a IVS3 +281 T allele (GT and TT genotype), had lower TAG concentrations than those with only a G allele; the estimated effect of any T allele was 82% of the GG genotype (*p* = 0.008, dominant model). The association was also significant in the combined group (Supplementary Information, Table S1), suggesting that this association was not different for the black and white women.

### 3.5. Dietary Intake

Dietary intake of the normal-weight and obese, black and white women are summarised in Table 3. Macronutrient and dietary fat intake are expressed as a percentage of total energy intake (%E). The obese groups consumed more energy (kJ), total fat, saturated fat (SFA), polyunsaturated fat (PUFA), omega-6 (*n*-6) PUFA, linoleic acid (LA), arachidonic acid (AA), eicosapentaenoic acid (EPA), and docosahexaenoic acid (DHA) (%E), than the normal-weight groups. The black women consumed more energy (kJ), carbohydrate (CHO), total fat, PUFA, *n*-6 PUFA, LA, AA, EPA, and DHA (%E), as well as had higher PUFA:SFA (P:S) and omega-6:omega-3 (*n*-6:*n*-3) ratios than the white women. In contrast, the white women consumed more protein, SFA and α-linolenic acid (ALA) (%E) compared to the black women.

**Table 3:** Dietary intakes of normal-weight and obese adequate reporter black and white women.

Dietary variable	Black		White		*p*-value	
	Normal-weight	Obese	Normal-weight	Obese	BMI Group	Ethnic Group
*n*	73	74	73	48	-	-
Fat (%E)	33.7 (28.3–37.2)	34.7 (31.1–38.5)	29.6 (25.7–32.5)	30.6 (26.3–33.9)	0.023	0.002
SFA (%E)	9.2 (7.9–10.7)	9.5 (8.3–10.9)	9.7 (8.3–12.0)	11.2 (9.1–12.3)	0.041	<0.001
MUFA (%E)	10.3 (8.8–11.8)	11.4 (9.8–12.9)	10.2 (8.7–11.6)	10.4 (8.4–12.1)	0.05	0.844
PUFA (%E)	8.4 (6.8–10.2)	9.5 (7.7–11.0)	6.1 (4.9–7.9)	6.1 (4.8–7.6)	0.007	<0.001
P:S ratio	0.91 (0.73–1.17)	1 (0.81–1.21)	0.62 (0.46–0.78)	0.56 (0.48–0.72)	0.205	<0.001
(*n*-3) PUFA (%E)	0.28 (0.22–0.34)	0.36 (0.27–0.46)	0.33 (0.26–0.41)	0.32 (0.27–0.39)	0.296	0.997
(*n*-6) PUFA (%E)	7.9 (6.6–10.3)	9.1 (7.3–10.7)	5.5 (4.2–7.5)	5.5 (4.4–7.2)	0.01	<0.001
(*n*-6):(*n*-3) PUFA ratio	27 (20.0–39.9)	25.7 (18.3–36.5)	15.9 (12.3–22.5)	16.4 (13.0–24.9)	0.203	<0.001
ALA (%E)	0.21 (0.18–0.25)	0.22 (0.18–0.27)	0.26 (0.21–0.30)	0.25 (0.21–0.29)	0.372	<0.001
LA (%E)	8.1 (6.70–10.6)	9.3 (7.50–10.9)	5.5 (4.20–7.40)	5.5 (4.40–7.20)	0.011	<0.001
AA (%E)	0.04 (0.030–0.060)	0.06 (0.040–0.070)	0.03 (0.020–0.040)	0.04 (0.030–0.050)	<0.001	<0.001
EPA (%E)	0.017 (0.009–0.035)	0.037 (0.016–0.065)	0.014 (0.009–0.023)	0.018 (0.009–0.025)	0.037	<0.001
DHA (%E)	0.037 (0.023–0.076)	0.079 (0.038–0.128)	0.047 (0.029–0.063)	0.045 (0.028–0.066)	0.043	0.001

IQR, interquartile range; SFA, saturated fatty acid; MUFA, monounsaturated fatty acid; PUFA, polyunsaturated fatty acid; P:S ratio, polyunsaturated fatty acid: saturated fatty acid ratio; (*n*-3) PUFA, omega-3 polyunsaturated fatty acid; (*n*-6) PUFA, omega-6 polyunsaturated fatty acid; (*n*-6):(*n*-3) PUFA ratio, omega-6: omega-3 polyunsaturated fatty acid ratio; ALA, α-linolenic acid; LA, linoleic acid; AA, arachidonic acid; EPA, eicosapentaenoic acid; DHA, docosahexaenoic acid. Dietary intake includes adequate reporters only. Summarised as median (interquartile range). Outcomes were log-transformed when required for modelling. *p*-values are from age-adjusted linear models of outcomes, testing the difference between BMI and ethnic groups each adjusted for the other.

### 3.6. Diet-Genotype Interactions on BMI and Body Composition

#### 3.6.1. *IL-6* −174 G>C

**Figure 4:**
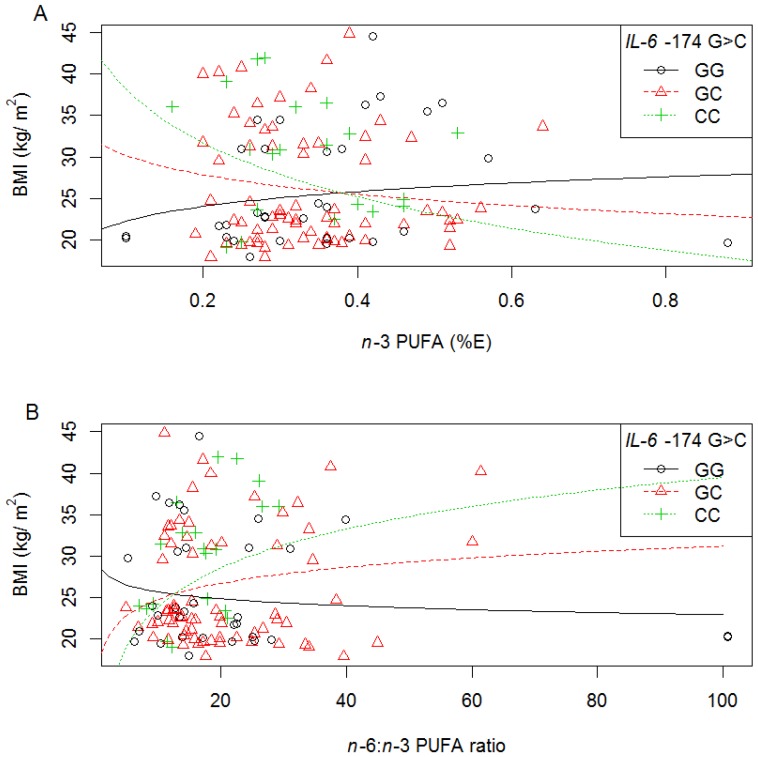
The relationship between BMI, *IL-6* −174 G>C polymorphism and dietary fat intake in white women. Symbols represent, for each woman, observed values. The lines are modeled relationships for a woman of average age (27.3 years). (**A**) With increasing *n*-3 PUFA intake (%E), BMI decreased in those with the −174 CC or GC genotypes (*R*^2^ = 0.17, *p* = 0.027); (**B**) With increasing *n*-6:*n*-3 PUFA ratio, BMI increased equally with each additional −174 C allele (*R*^2^ = 0.8, *p* = 0.028).

In white women, interactions between the *IL-6* −174 G>C polymorphism and the intake of different dietary fatty acids (%E) on BMI were found (Tables S2 and S3, and summarised in Table 4. With increasing total *n*-3 PUFA (*p* = 0.027) (Figure 4A), EPA (*p* = 0.040) and DHA (*p* = 0.043) intake (%E), a decrease in BMI was observed in individuals with a −174 C allele (CC or GC genotypes, dominant). In addition, as the *n*-6:*n*-3 PUFA ratio increased, BMI increased equally with each additional −174 C allele (additive, *p* = 0.028) (Figure 4B). Diet-genotype interactions, as well as the impact of ethnicity for the −174 G>C polymorphism on body composition were not analysed due to the rare frequency of the C allele in the black women.

**Table 4:** Summary of interleukin 6 (*IL-6*) diet-gene interactions on body composition.

Dietary variable	SNP	Black					White			
		Weight, kg	BMI, kg/m^2^	Fat Mass, kg	Waist, cm	BMI, kg/m^2^	Fat Mass, kg	Body Fat %	Waist, cm	WHR
Total fat	IVS3 +281 G>T	↑ GG or GT ↓ TT	↑ GG or GT ↓ TT	↑ GG or GT ↓ TT	↑ GG or GT ↓ TT	-	-	-	-	-
	IVS4 +869 A>G	↑ AG or AA ^a^ ↓ GG ^a^	↑ AG or AA ^a^ ↓ GG ^a^	↑ AG or AA ^a^ ↓ GG ^a^	↑ AG or AA ↓ GG ^a^	-	-	-	-	-
SFA	IVS3 +281 G>T	-	↑ GG or G ^b^ ↓ TT ^b^	↑ GG or GT ↓ TT	-	-	-	-	-	-
	IVS4 +869 A>G	↑ AG or AA ^a^ ↓ GG ^a^	↑ AG or AA ^a^ ↓ GG ^a^	↑ AG or AA ^a^ ↓ GG ^a^	↑ AG or AA ^a^ ↓ GG ^a^	-	-	-	-	-
MUFA	IVS3 +281 G>T	-	↓ TT	↑ GG or GT ↓ TT	-	-	-	-	-	-
	IVS4 +869 A>G	↑ AG or AA ^a^ ↓ GG ^a^	↑ AG or AA ^a^ ↓ GG ^a^	↑ AG or AA ^a^ ↓ GG ^a^	↑ AG or AA ^a^ ↓ GG ^a^	-	-	-	-	-
PUFA	IVS3 +281 G>T	↑ GG or GT ↓ TT	↑ GG or GT ^b^ ↓ TT ^b^	↑ GG or GT ↓ TT	↑ GG or GT ↓ TT	-	-	-	-	-
*n*-6 PUFA	IVS3 +281 G>T	↑ GG or GT ↓ TT	↑ GG or G ^b^ ↓ TT ^b^	↑ GG or GT ↓ TT	-	-	-	-	-	-
LA	IVS3 +281 G>T	↑ GG or GT ↓ TT	↑ GG or G ^b^ ↓ TT ^b^	↑ GG or GT ↓ TT	-	-	-	-	-	-
AA	IVS3 +281 G>T	↑ GG or GT ↓ TT	↑ GG or GT ^b^ ↓ TT ^b^	↑ GG or GT ↓ TT	-	-	-	-	-	-
	IVS4 +869 A>G	↑ AG or AA	↑ AG or AA	↑ AG or AA	-	-	-	-	-	-
*n*-3 PUFA	−174 G>C	-	-	-	-	↓ C allele	-	-	-	-
	IVS4 +869 A>G	-	-	-	-	-	↓ AG or GG	-	-	-
EPA	−174 G>C	-	-	-	-	↓ C allele	-	-	-	-
DHA	−174 G>C	-	-	-	-	↓ C allele	-	-	-	-
ALA	IVS3 +281 G>T	-	-	-	-	↓ T allele	-	-	↓ T allele	↓ T allele
	IVS4 +869 A>G	-	-	-	-	-	↓ AG or GG ^b^ ↑ AA ^ab^	-	-	-
*n*-6:*n*-3 ratio	−174 G>C	-	-	-	-	↑ C allele	-	-	-	-
	IVS3 +281 G>T	-	-	-	-	↑ T allele ^b^	-	↑ T allele	↑ T allele ^b^	-
	IVS4 +869 A>G	-	-	-	-	-	↑ AG or GG ^c^	-	-	-

The interactions are described as follows: With increasing dietary fat variable, the body composition outcome either ↑ (increases) or ↓ (decreases) for a particular genotype or allele. Where there was no interaction, the data is excluded. All interactions are diet-genotype unless stated as being allelic. Dietary fat intake is calculated as a percentage of total energy intake. ^a^ These interactions were significant but the individual rates of change were not significant; ^b^ These interactions were ethnic specific; ^c^ This interaction was not different for black and white women. Abbreviations: WHR, waist:hip ratio; SFA, saturated fatty acid; MUFA, monounsaturated fatty acid; PUFA, polyunsaturated fatty acid; *n*-3 PUFA, omega-3 polyunsaturated fatty acid; *n*-6 PUFA, omega-6 polyunsaturated fatty acid; *n*-6:*n*-3 PUFA ratio, omega-6: omega-3 polyunsaturated fatty acid ratio; ALA, α-linolenic acid; LA, linoleic acid; AA, arachidonic acid; EPA, eicosapentaenoic acid; DHA, docosahexaenoic acid.

#### 3.6.2. IVS3 +281 G>T

**Figure 5:**
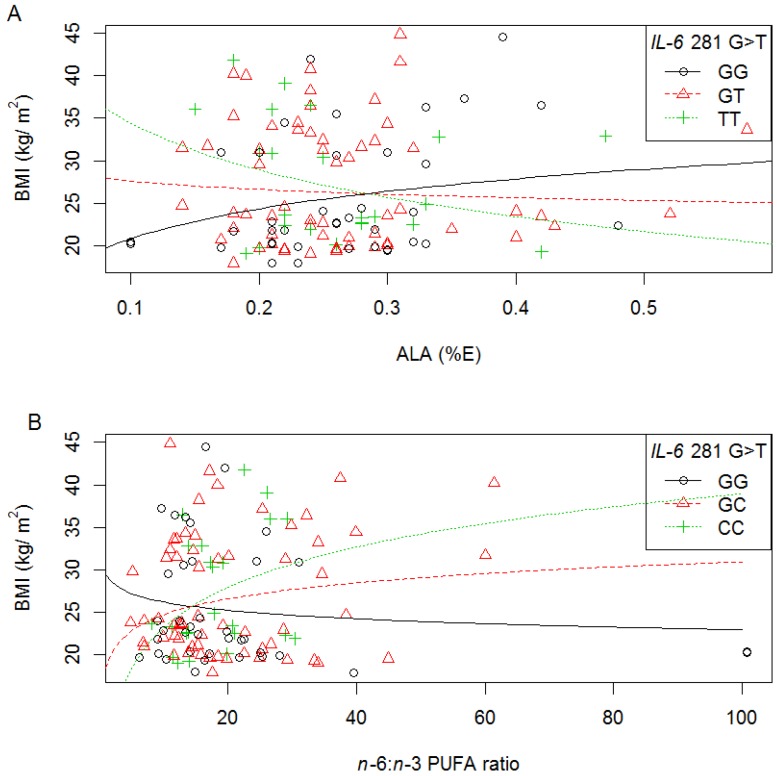
The relationship between BMI, *IL-6* IVS3 +281 G>T polymorphism and dietary fat intake in white women. Symbols represent, for each woman, observed values. The lines are modeled relationships for a woman of average age (27.3 years). (**A**) With increasing ALA intake (%E), BMI decreased with each additional IVS3 +281 T allele (*R*^2^ = 0.16, *p* = 0.032); (**B**) With increasing *n*-6:*n*-3 PUFA ratio, BMI increased with each additional IVS3 +281 T allele (*R*^2^ = 0.18, *p* = 0.030).

Interactions were also observed in white women between IVS3 +281 G>T and ALA intake (%E) on BMI (*p* = 0.032), waist (*p* = 0.038) and WHR (*p* = 0.037), as well as between IVS3 +281 G>T and *n*-6:*n*-3 PUFA ratio on BMI (*p* = 0.030), waist (*p* = 0.048), and body fat % (*p* = 0.044) (Tables S2 and S3, and summarised in Table 4.). More specifically, with increasing ALA intake (%E), BMI decreased with each T allele (Figure 5A); while with increasing *n*-6:*n*-3 PUFA ratio, BMI increased with each additional IVS3 +281 T allele (additive models) (Figure 5B). In all cases, the rate of change (increase and decrease) was significant for the minor homozygotes, TT. Similar to the −174 G>C interactions described above, slopes were in the opposite direction depending on the fatty acids consumed.

In black women, we also identified interactions between IVS3 +281 G>T and dietary fat intake (*p* = 0.006), PUFA (*p* = 0.004), SFA (*p* = 0.010), monounsaturated fat (MUFA) (*p* = 0.046), *n*-6 PUFA (*p* = 0.004), LA (*p* = 0.006), and AA (*p* = 0.005), intake (%E) on BMI (Tables S2 and S3, and summarized in Table 4). With increasing dietary fat intake, BMI decreased in those with the IVS3 +281 TT genotype and increased in those with the GG or GT genotype (recessive). With increasing MUFA intake (*p* = 0.046), BMI also decreased in those with the IVS3 +281 TT genotype, but the increase in those with the GG or GT genotype was not significant. Similar effects were detected for weight (total dietary fat (*p* = 0.012), PUFA (*p* = 0.007), *n*-6 PUFA (*p* = 0.009), LA (*p* = 0.009) and AA (*p* = 0.008)), waist (for total dietary fat (*p* = 0.019) and PUFA (*p* = 0.013)), and for fat mass (total fat (*p* = 0.005), SFA (*p* = 0.010), MUFA (*p* = 0.013), PUFA (*p* = 0.004), *n*-6 PUFA (*p* = 0.005), LA (*p* = 0.006), and AA (*p* = 0.004)). For all these interactions, with increasing dietary fat intake, adiposity decreased in those with the IVS3 +281 TT genotype and increased in those with the GG or GT genotype.

The ethnic-specific nature of these diet-genotype interactions was identified by including both black and white women in the same model and adjusting for ethnicity (Table S4 (genotype) and S5 (allelic)). As well as by examining three-way interactions between ethnic group, dietary fat intake (%E) and the *IL-6* polymorphisms on body composition (Tables S6 (genotype) and S7 (allelic)).

Tables S6 (genotype) and S7 (allelic), which report the *p*-values for three-way interactions between ethnic group, dietary fat intake (%E) and the IVS3 +281 G>T polymorphism on body composition, identified significant interactions between ethnicity and SFA (*p* = 0.045), PUFA (*p* = 0.024), *n*-6 PUFA (*p* = 0.027), LA (*p* = 0.029), AA (*p* = 0.005), and *n*-6:*n*-3 PUFA ratio (*p* = 0.023) and the IVS3 +281 G>T genotypes on BMI. Interactions between ethnicity, *n*-6:*n*-3 PUFA ratio and IVS3 +281 G>T genotypes (*p* = 0.015) and alleles (*p* = 0.049) on waist were also observed. This suggests that these interactions, reported above, were different between the black and white women. Significant diet-gene interactions identified for the IVS3 +281 G>T polymorphism, but not found to be significant in the three-way interactions, were also not significant in the combined group (Table S4 (genotype) and S5 (allelic)).

#### 3.6.3. IVS4 +869 A>G

**Figure 6:**
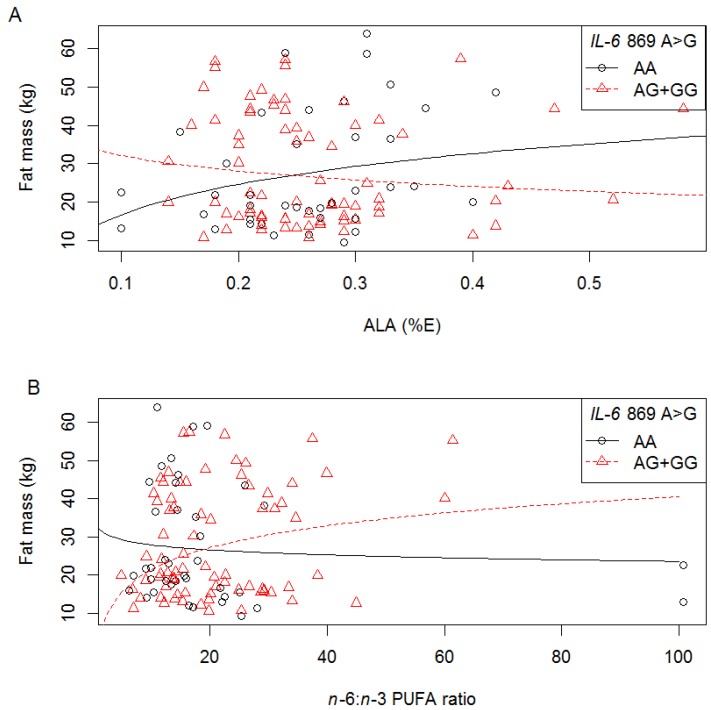
The relationship between fat mass, *IL-6* IVS4 +869 A>G polymorphism and dietary fat intake in white women. Symbols represent, for each woman, observed values. The lines are modeled relationships for a woman of average age (27.3 years). (**A**) With increasing ALA intake (%E), fat mass decreased in those with the IVS4 +869 AG or GG genotype (*R*^2^ = 0.13, *p* = 0.048); (**B**) With increasing *n*-6:*n*-3 PUFA ratio, fat mass increased in those with the IVS4 +869 AG or GG genotype; compared to those with the AA genotype (*R*^2^ = 0.15, *p* = 0.034).

In white women, we also detected interactions between IVS4+869 A>G polymorphism and ALA (%E) (*p* = 0.048, Figure 6A), *n*-3 PUFA (%E) (*p* = 0.034) and *n*-6:*n*-3 PUFA ratio (*p* = 0.034, Figure 6B) on fat mass (Tables S2 and S3, and summarised in Table 4.). With increasing *n*-3 PUFA (%E) intake, fat mass decreased in those with AG or GG genotype, while with increasing *n*-6:*n*-3 PUFA ratio, fat mass increased in those with an AG+GG genotype; compared to those with the AA genotype (Figure 6B). With increasing ALA (%E) intake, fat mass increased in those with the AA genotype and decreased in those with the AG+GG genotype, however the individual rates were not significant (Figure 6A). These interactions are similar to −174 G>C and IVS3 +281 G>T in the white women where opposite slopes were observed for *n*-3 PUFA and the *n*-6:*n*-3 PUFA ratio.

In black women, we also observed a number of diet-gene interactions with the IVS4 +869 A>G polymorphism on adiposity (Tables S2 and S3, and summarised in Table 4), which where similar to interactions shown for the IVS3 +281 G>T polymorphism. With increasing total dietary fat (*p* = 0.009), MUFA (*p* = 0.010), and SFA (*p* = 0.007) intake (%E), BMI, (as well as weight, waist and fat mass) increased in those with the IVS4 +869 AA+AG genotype and decreased in those with the IVS4 +869 GG genotype. However, the individual rates of change were not significant. With increasing AA intake (%E), BMI (*p* = 0.010), weight (*p* = 0.016) and fat mass (*p* = 0.014) increased for the AA+AG genotypes (recessive effects), but were not significant for the GG genotype.

Tables S6 (genotype) and S7 (allelic), which reports the p-values for three-way interactions between ethnic group, dietary fat intake (%E) and the IVS4 +869 A>G polymorphism on body composition, identified significant interactions between ethnicity, ALA intake and the IVS4 +869 A>G genotypes (*p* = 0.010) and alleles (*p* = 0.036) on fat mass. This suggests that these interactions, reported above, were different between the black and white women.

When the black and white women were combined and adjusted for age and ethnicity (Tables S4 and S5), the interactions between total fat (%E) and SFA (%E) and the IVS4 +869 A>G polymorphism on weight, BMI, waist and fat mass were significant (*p* = 0.009, *p* = 0.029, *p* = 0.011, and *p* = 0.018, for total fat intake respectively, and *p* = 0.014, *p* = 0.009, *p* = 0.015, and *p* = 0.049, for SFA intake respectively). These interactions were only significant in the black women. In the combined group an interaction between *n*-6:*n*-3 PUFA ratio and IVS4 +869 A>G polymorphism on fat mass was significant (*p* = 0.035), but this was only observed in the white women. This suggests that these interactions identified in the combined group were not different for the black and white women.

### 3.7. Diet-Genotype Interactions on Serum Lipid Concentrations

#### 3.7.1. IL-6 −174 G>C

In white women, we detected interactions between −174 G>C polymorphism and the intake of different dietary fatty acids (%E) on T-C, TAG, HDL-C, and T-C:HDL-C ratio (Tables S8 and S9, and summarized in Table 5). With increasing MUFA (*p* = 0.015), and EPA (*p* = 0.044) intakes, TAG decreased with each –174 C allele, and with increasing *n*-3 PUFA (*p* = 0.047) and ALA (*p* = 0.022) intakes (%E), HDL-C increased with each −174 C allele (additive effects). With increasing EPA and DHA intakes (%E) (*p* = 0.021 and *p* = 0.014, respectively), the T-C:HDL-C ratio decreased in those with the −174 CC compared to CG+GG genotype (recessive effect). Diet-genotype interactions, as well as the impact of ethnicity for the −174 G>C polymorphism on serum lipids were not analysed due to the rare frequency of the C allele in the black women.

**Table 5:** Summary of *IL-6* diet-gene interactions on serum lipids.

Dietary variable	SNP	Black			White		
		TAG, mmol/L	LDL-C, mmol/L	T-C:HDL-C Ratio	TAG, mmol/L	HDL-C, mmol/L	T-C:HDL-C Ratio
**Total fat**	**IVS3 +281 G>T**	-	↓ VGT	↓ GT	-	-	-
	**IVS4 +869 A>G**	↑ GG ↓ AA	-	↑ GG ^a^ ↓ AA ^a^	-	-	-
**MUFA**	**−174 G>C**	-	-	-	↓ C allele	-	-
	**IVS3 +281 G>T**	-	↓ GT	↓ GT	-	-	-
**PUFA**	**IVS3 +281 G>T**	*-*	↓ GT ^b^	↓ GT	-	-	-
***n* -6 PUFA**	**IVS3 +281 G>T**	-	↓ GT ^b^	↓ GT	-	-	-
**LA**	**IVS3 +281 G>T**	-	↓ GT ^b^	↓ GT	-	-	-
***n* -3 PUFA**	**−174 G>C**	-	-	-	-	↑ C allele	-
**EPA**	**−174 G>C**	-	-	-	↓ C allele	-	↓ CC
	**IVS3 +281 G>T**	-	-	-	-	-	↓ TT
	**IVS4 +869 A>G**	-	-	-	-	↓ G allele ^a^	-
**DHA**	**−174 G>C**	-	-	-	-	↑ C allele	↓ CC
	**IVS4 +869 A>G**	-	-	-	-	↓ G allele ^a^	-
**ALA**	**−174 G>C**	-	-	-	-	↑ C allele	-
	**IVS3 +281 G>T**	-	-	-	-	↑ T allele	↓ TT ^b^
***n* -6: *n* -3 ratio**	**IVS4 +869 A>G**	↑ GG ↓ AA	-	↑ GG ↓ AA	-	-	-

The interactions are described as follows: With increasing dietary fat variable, the serum lipid outcome either ↑ (increases) or ↓ (decreases) for a particular genotype or allele. Where there was no interaction, the data is excluded. All interactions are diet-genotype unless stated as being allelic. Dietary fat intake is calculated as a percentage of total energy intake. ^a^ These interactions were ethnic specific; ^b^ These interactions were not different for black and white women. Abbreviations: TAG, triglycerides; T-C, total cholesterol; HDL-C, high density lipoprotein cholesterol; LDL-C, low density lipoprotein cholesterol; T-C:HDL-C ratio, total cholesterol: high density lipoprotein cholesterol ratio, SFA, saturated fatty acid; MUFA, monounsaturated fatty acid; PUFA, polyunsaturated fatty acid; P:S ratio, polyunsaturated fatty acid: saturated fatty acid ratio; *n*-3 PUFA, omega-3 polyunsaturated fatty acid; *n*-6 PUFA, omega-6 polyunsaturated fatty acid; *n*-6:*n*-3 PUFA ratio, omega-6: omega-3 polyunsaturated fatty acid ratio; ALA, α-linolenic acid; LA, linoleic acid; EPA, eicosapentaenoic acid.

#### 3.7.2. IVS3 +281 G>T

In white women, with increasing *n*-3 PUFA (*p* = 0.015), and ALA (*p* = 0.029) intakes (%E), T-C:HDL-C ratio decreased only in those with the IVS3 +281 TT genotype compared to TG+GG genotype (recessive), and with increasing ALA intake (%E), HDL-C increased significantly with each additional IVS3 +281 T allele (*p* = 0.018) (Tables S8 and S9, and summarized in Table 5).

In black women, the interactions between IVS3 +281 G>T and the intake of dietary fatty acids on serum lipids were unusual, in that with increasing dietary fat intake (%E) (Fat, MUFA, PUFA, *n*-6 PUFA and LA), the serum lipid levels (LDL-C and T-C:HDL-C ratio) decreased in those with a GT genotype, but there was no effect (increasing, but no statistical significance was observed) in those who were homozygous for either allele.

The ethnic-specific nature of these diet-genotype interactions was investigated by including both black and white women in the same model and adjusting for ethnicity (Table S10 (genotype) and Table S11 (allelic)). As well as by examining three-way interactions between ethnic group, dietary fat intake (%E) and the *IL-6* polymorphisms on serum lipids (Tables S12 (genotype) and S13 (allelic).

No diet-gene interactions were identified in the three-way interactions on serum lipids (Tables S12 and S13). When the black and white women were combined and adjusted for age and ethnicity (Tables S10 and S11), the interactions between PUFA (%E), *n*-6 PUFA (%E) and LA (%E) intake and IVS3 +281 G>T genotypes on LDL-C were significant (*p* = 0.021, *p* = 0.026, and *p* = 0.026, respectively), and between ALA intake and IVS3 +281 G>T genotypes on the T-C:HDL-C ratio (*p* = 0.027). Of these, the interactions on LDL-C were only significant in the black women and the interaction on the T-C:HDL-C ratio was significant in the white women. This suggests that these interactions described above, and identified in the combined group were not different for the black and white women.

#### 3.7.3. IVS4 +869 A>G

In white women, with increasing EPA and DHA (%E) intakes (*p* = 0.047 and *p* = 0.023, respectively), HDL-C decreased with each IVS4+869 G allele (additive); in those with the minor homozygote GG genotype, the decrease in HDL-C was significant (Tables S8 and S9, and summarized in Table 5.).

In black women, an interaction was observed between increasing total dietary fat intake (%E) and *n*-6:*n*-3 PUFA ratio, and TAG (*p* = 0.049), and T-C:HDL-C ratio (*p* = 0.029) respectively, with each IVS4 +869 G allele. With increasing dietary fat intake, a decrease in serum lipids was noted in individuals who were homozygous AA, compared to increases in serum lipids in individuals who were homozygous GG.

Table S13 (allelic), which reports the *p*-values for three-way interactions between ethnic group, dietary fat intake (%E) and the IVS3 +281 G>T polymorphism on serum lipids, identified significant interactions between EPA (*p* = 0.026) and DHA (*p* = 0.013), and the IVS4 +869 A>G alleles on HDL-C reported above, suggesting that these interactions are different between the black and white women.

When the black and white women were combined and adjusted for age and ethnicity (Tables S10 and S11), only the interaction between total fat (%E) and IVS4 +869 A>G polymorphism on the T-C:HDL-C ratio was significant (*p* = 0.013) (Table S10), and only in the black women, suggesting that it is different between the black and white women.

## 4. Discussion

This study identified associations between the G allele of the *IL-6* IVS4 +869 A>G polymorphism and increasing adiposity in the black women, and the T allele of the *IL-6* IVS3 +281 G>T polymorphism and lower TAG concentrations in the white women. For the first time, we also showed that dietary fatty acids modulate the relationship between *IL-6* polymorphisms and measures of obesity and serum lipids in both black and white SA women. *A priori* our hypothesis, the −174 G>C, IVS4 +869 A>G and IVS3 +281 G>T polymorphisms in white women, and the latter two intronic polymorphisms in black women were in strong LD, and showed similar diet-gene interactions for their minor alleles, but these relationships differed between the black and white women.

Previous studies have shown the C allele of the *IL-6* −174 G>C polymorphism was associated with increased adiposity [13], and the T allele of the IVS3 +281 G>T polymorphism was associated with adiposity when part of a haplotype [10]. However, in agreement with previous studies in black populations [25,41,42], the black SA women had a very low −174 C allele frequency, which may explain why no association was found with this allele. It is also interesting to note that black SA women have a high prevalence of obesity [43], greater abdominal and peripheral SAT [44], and a higher SAT inflammatory gene expression profile compared to white SA women [45], potentially explaining why the associations between *IL-6* IVS4 +869 A>G genotypes and adiposity was only observed in black, but not white women. Notably, the findings of the present study are supported by our previous studies examining polymorphisms within the pro-inflammatory cytokine gene, *TNFA*. In these studies we found that for both the *TNFA* −308 G>A [17,18] and −238 G>A polymorphisms [19], the pro-inflammatory −308 A allele and the −238 A allele were associated with obesity risk and adiposity in black, but not white SA women.

Conversely, and in line with our previous publications, the association between the IVS3 +281 G>T polymorphism and lower TAG concentrations was found only in white women. White SA women have a more atherogenic lipid profile than black women [46], which may be related to their higher levels of VAT [47]. Limited data is available concerning the role of *IL-6* polymorphisms in lipid metabolism [16,48] in Caucasian and African American populations [48,49,50]. Most studies, but not all [16], found −174 G allele carriers to have higher T-C, LDL-C and TAG concentrations, [6,8,15] and lower HDL-C concentrations [8,16]. Henningson *et al.* reported the −174 CC genotype was associated with lower TAG, T-C and LDL-C concentrations in Swedish women, however in men, C allele carriers displayed elevated TAG concentrations [6]. In contrast, Riikola *et al.* reported that T-C and LDL-C concentrations were higher in Finnish men with the –174 GG genotype compared to the CG and CC genotypes, but not in women. These conflicting results highlight the complexity of these associations [8,9].

The first SA Demographic Health Survey (SADHS, 2003), found the overall prevalence of overweight and obesity in South African women to be high, with black women being more affected than white women (58.5% *vs.* 52.9%) [43]. The recent 2013 South African National Health and Nutrition Examination Survey (SANHANES-1) found overweight and obesity in black women to have increased to 64.8% [51]. Obesity in black women is associated with a higher prevalence of insulin resistance and type 2 diabetes, whereas obese white women traditionally have a higher prevalence of dyslipidaemia and associated CVD [52]. As ethnicity encompasses genetic, environmental, lifestyle (including diet) and cultural differences, and given that diet and environment contribute significantly to the pathogenesis and development of these chronic diseases, it is unlikely that genetic differences alone would underlie these ethnic differences [53]. The black population in SA has undergone a rapid diet transition from a rural diet, high in complex carbohydrates, to a more westernised urban intake, high in fat [54], with subsequent increases in the prevalence of diseases of lifestyle [52]. In contrast, polymorphism frequencies are unlikely to change in this time span. We therefore hypothesize that individuals may take different routes in their development of obesity and co-morbidities, and that the quantity and quality of dietary fat intake (as well as other environmental and lifestyle factors), and the relative frequency of genetic variants may impact the obese phenotype.

In addition to the associations reported here, this study shows for the first time that both the quality and quantity of dietary fat intake modulates this relationship. When dietary fat intake was included in the analyses; in the white women, with increasing intake of the anti-inflammatory *n*-3 PUFAs, measures of adiposity decreased, and with increasing *n*-6:*n*-3 PUFA ratio, adiposity increased in those with the −174 C, IVS3 +281 T and IVS4 +869 G minor alleles. These findings are supported by Razquin *et al.* who showed that with increasing *n*-3 PUFA intake, the −174 CC genotype was associated with lower measures of adiposity [24]. Further, in white women, we showed that an increasing intake of *n*-3 PUFAs was associated with a decrease in TAG (only for −174 G>C) and T-C:HDL-C ratio, and an increase in HDL-C in those with the −174 C and IVS3 +281 T alleles. These findings suggest that anti-inflammatory *n*-3 PUFAs have a favourable effect on adiposity and serum lipids, but only in individuals with the −174 C, IVS3 +281 T, and IVS4 +869 G minor alleles.

In contrast, in the black women it appears that an increasing intake of dietary fat, rather than the quality of individual fatty acids, was associated with increasing measures of adiposity in those with the IVS3 +281 GT+GG and IVS4 +869 AG+AA genotypes, and decreasing measures of adiposity in those with the IVS3 +281 TT and IVS4 +869 GG genotypes. Further, with increasing total fat intake and *n*-6:*n*-3 ratio, TAG and T-C:HDL-C ratio increased with each IVS4 +869 G allele. In previous studies of the *TNFA* −308 G>A and −238 G>A polymorphisms [17,18,19] in the same population, we also observed that in black women, total dietary fat intake was associated with greater measures of obesity for the −308 and −238 A alleles irrespective of the quality of the dietary fat.

It has been proposed that dietary fatty acids affect inflammatory processes through modulation of eicosanoid metabolism and eicosanoid-independent mechanisms, influencing transcription factors involved in inflammation [55,56]. Oxidised lipids, SFAs and *trans* fatty acids have been shown to promote inflammation, and *n*-3 PUFAs, especially marine *n*-3 PUFAs are protective against inflammation [57]. Controversy exists as to the inflammatory affect of the different *n*-6 PUFAs [57], and a high *n*-6:*n*-3 PUFA ratio is generally considered to be associated with inflammation [58], but not consistently so [57].

Studies have shown that dietary fatty acids modulate IL-6 production, thereby influencing inflammatory status [59]. A number of studies have investigated the effect of different dietary fatty acids on IL-6 levels in different models, with human studies showing similar results to cell culture and rodent models [59]. He *et al.* showed that increased *n*-3 PUFA intake and fish consumption were associated with decreased plasma concentrations of IL-6 and other inflammatory markers [60]. Further, Ferrucci *et al.* reported that plasma levels of PUFAs, especially *n*-3 PUFAs, were independently associated with lower levels of pro-inflammatory markers and higher levels of anti-inflammatory markers [61]. Rats fed a diet high in *n*-6 PUFA-rich sunflower oil showed a moderate IL-6 release from adipocytes, and lower plasma IL-6 than when fed a SFA-rich diet, but greater than when fed a MUFA-rich diet [62]. Only one controlled feeding trial investigated the effect of a SFA and MUFA-rich diet on serum lipid concentrations and whole-genome microarray gene expression profiles of adipose tissue [63]. The eight-week consumption of a SFA-enriched diet resulted in increased expression of genes involved in inflammatory processes in adipose tissue, including IL-6 and NF-κB signalling, whereas the MUFA enriched diet led to a more anti-inflammatory gene expression profile, accompanied by a decrease in serum LDL-C concentration [63].

Unfortunately, we were not able to measure circulating IL-6 or other inflammatory markers (e.g., hs-CRP) in this study. However, the published findings support our study hypothesis that dietary fatty acids may influence obesity risk and serum lipid levels differently, depending on the *IL-6* genotype of the individual. For the *IL-6* −174 G>C, IVS3 +281 G>T, and IVS4 +869 A>G polymorphisms reported here it appears that the minor alleles of these polymorphisms may be more responsive to the quantity and quality of the dietary fat being consumed, compared to the major allele. Similar results have been observed for the *TNFA* polymorphisms, where the minor A allele was more responsive to changes in dietary fat intake [17,18]. Notably, in this study the individual diet-gene interactions differed between the two ethnic groups. The reasons for this are not known, but may relate to differences in the frequency of the polymorphisms (Table 2) and/or differences in dietary intake (Table 3), specifically dietary fatty acid intake, between black and white women included in this study.

The minor allele frequencies of the −174 G>C and IVS3 +281 G>T polymorphisms were higher in the white compared to the black women, but all frequencies were similar to those from European and other African populations. In this study the diet-gene interactions in the white women, for both body composition and serum lipids, were observed only with regards the *n*-3 PUFA’s and the *n*-6:*n*-3 PUFA ratio, whereas in the black women, the diet-gene interactions were observed for total fat intake, SFA, MUFA and PUFA, and for all the *n*-6 fatty acids. The intake of *n*-6 PUFAs (%E) and the *n*-6:*n*-3 PUFA ratio in the black women is alarmingly high, and higher than that of the white women (8.3% *vs.* 5.6%, and 26.4:1 *vs.* 16.1:1, for black and white women, respectively), and that reported in other populations, 4.0%–6.0% for *n*-6 PUFA (%E) [64], and ≈15:1 for the *n*-6:*n*-3 PUFA ratio [65].

While the functional nature of the −174 G>C polymorphism has been investigated, little is known of the functional nature of the two additional intronic polymorphisms included in this study, especially their impact on markers of inflammation. Unfortunately, circulating IL-6 levels were not measured in this study. Furthermore, other polymorphisms, such as those found on the fatty acid desaturase 1 (*FADS1*) and *FADS2* genes, may alter PUFA metabolism and these gene-gene interactions may need to be considered [66]. It is also important to consider the study design. The GWAS approach has yielded many successful discoveries [67], identifying at least 50 loci associated with BMI, WHR, body fat percentage, and extreme obesity [68], as well as genetic variants associated with variation in serum lipid concentrations [69]. However, no GWAS studies have found *IL-6* genetic variants to be associated with obesity measures in Caucasian or African American populations [70]. A further limitation of GWAS in the context of this study is that they have been conducted primarily in white European populations, with only one study including a population from the African continent and none from Southern Africa [71]. Notably, GWAS have also not to date been used to address gene-diet and gene-environment interactions, which are essential to our understanding of how diet and environment may modulate genotype-phenotype associations identified.

Further limitations to this study should be considered. In brief, our sample size was small and included fewer white than black women. It is always difficult to report accurate dietary intake. To improve the validity of the dietary intake data we used a validated FFQ developed specifically for the SA population, and included only adequate reporters in the analyses. No lifestyle factors such as physical activity and socioeconomic factors were included, that may also impact the obese phenotype.

## 5. Conclusions

In conclusion, this novel study showed that dietary fat intake, and the quality of the dietary fatty acids consumed, were associated with obesity risk and serum lipids differently, depending on the *IL-6* genotype. Notably, these effects were different in black and white women. For the first time this study showed that two *IL-6* polymorphisms (IVS3 +281 G>T and IVS4 +869 A>G) that were informative as markers of gene-diet interactions in both black and white SA women were identified. Although the interaction between the *IL-6* genotype and dietary fatty acids only accounted for less than 20% of the variance in measures of body fatness, this study highlights the importance of understanding the inflammatory impact of different dietary fatty acids and how they may differentially interact with genes in different ethnic populations. Randomized controlled trials are required to examine the effects of specific dietary fatty acids on obesity risk and serum lipids in individuals with a specific *IL-6* (or other inflammatory cytokine) genotype. However, in order to make these findings generalizable, these studies should be performed in men and women, as well as individuals of different ethnicities.

## Supplementary Files

Supplementary File 1 Supplementary Information (DOCX, 120 KB)

## Abbreviations

AA: arachidonic acid
ALA: α-linolenic acid
CVD: cardiovascular disease
CHO: carbohydrate
DHA: docosahexaenoic acid
EPA: eicosapentaenoic acid
HDL-C: high density lipoprotein-cholesterol
hsCRP: high sensitivity C-reactive protein
HOMA-IR: homeostasis model assessment of insulin resistance
IL-6: interleukin 6
*IL-6*: interleukin six gene
LA: linoleic acid
LDL-C: low density lipoprotein-cholesterol
MUFA: mono-unsaturated fat
%E: percentage energy
(*n*-6) PUFA: (omega-6) polyunsaturated fatty acids
(*n*-3) PUFA: (omega-3) polyunsaturated fatty acid
(*n*-6):(*n*-3) PUFA ratio: (omega-6):(omega-3) polyunsaturated fatty acids ratio
P:S ratio: polyunsaturated:saturated fat ratio
SFA: saturated fat
SNP: single nucleotide polymorphism
SA: South African
T-C: total cholesterol
T-C:HDL-C ratio: total cholesterol:high density lipoprotein-cholesterol ratio
TAG: triacylglycerol
VAT: visceral adipose tissue
VLDL-C: very low density lipoprotein-cholesterol
WHR: waist hip ratio
