# Supplementary material for: Interleukin-6 Gene Polymorphisms, Dietary Fat Intake, Obesity and Serum Lipid Concentrations in Black and White South African Women
Source: Nutrients. 2014 Jun 24;6(6):2436–65. doi: 10.3390/nu6062436 (PMC4073161; doi:10.3390/nu6062436)
Supplement: Supplementary File 1 — Supplementary Information (DOCX, 120 KB) [file nutrients-06-02436-s001.docx]

Supplementary Information

**Abbreviations:** BMI, body mass index, HDL-C, high-density lipoprotein cholesterol; IL-6, Interleukin-6 gene; LDL-C, low-density lipoprotein cholesterol; SAT, subcutaneous adipose tissue; TAG, triacylglycerol; T-C, total cholesterol; T-C:HDL-C ratio, total cholesterol:HDL-cholesterol ratio; VAT, visceral adipose tissue; WHR, waist hip ratio. AA, arachidonic acid; ALA, α-linolenic acid; BMI, body mass index; DHA, docosahexaenoic acid; EPA, eicosapentaenoic acid, IL-6, Interleukin-6 gene; LA, linoleic acid; MUFA, mono-unsaturated fat; percentage energy; %E; PUFA, polyunsaturated fatty acid; *n*-6 PUFA, omega-6 polyunsaturated fatty acids; *n*-3 PUFA, omega-3 polyunsaturated fatty acid; *n*-6:*n*-3 PUFA ratio, omega-6:omega-3 polyunsaturated fatty acids ratio; P:S ratio, polyunsaturated:saturated fat ratio; SFA, saturated fatty acid; SAT, subcutaneous adipose tissue; VAT, visceral adipose tissue; WHR, waist hip ratio.

**Table S1.** *p*-values for black and white adequate reporter women combined, testing association with ethnic group, *IL-6* polymorphisms (allelic) and BMI group, on body composition and serum lipids, each adjusted for age and the factors listed before it.

| **Outcome** | ***IL-6* −174 G>C** | | | ***IL-6* IVS3 +281 G>T** | | | ***IL-6* IVS4 +869 A>G** | | |
| --- | --- | --- | --- | --- | --- | --- | --- | --- | --- |
|  | **Ethnic Group** | **BMI Group** | **−174** | **Ethnic Group** | **BMI Group** | **IVS3 +281** | **Ethnic Group** | **BMI Group** | **IVS4 +869** |
| *Body composition* | | | | | | | | | |
| Weight, kg | 0.065 | <0.001 | 0.319 | 0.016 | <0.001 | 0.037 | 0.039 | <0.001 | 0.377 |
| BMI, kg/m^2^ | <0.001 | <0.001 | 0.380 | <0.001 | <0.001 | 0.147 | <0.001 | <0.001 | 0.376 |
| Body fat, % | <0.001 | <0.001 | 0.369 | <0.001 | <0.001 | 0.827 | <0.001 | <0.001 | 0.848 |
| Fat Mass | <0.001 | <0.001 | 0.471 | <0.001 | <0.001 | 0.160 | <0.001 | <0.001 | 0.079 |
| Waist, cm | 0.012 | <0.001 | 0.473 | 0.005 | <0.001 | 0.113 | 0.012 | <0.001 | 0.278 |
| WHR | 0.168 | <0.001 | 0.804 | 0.174 | <0.001 | 0.460 | 0.151 | <0.001 | 0.339 |
| VAT, cm^2^ | <0.001 | <0.001 | 0.173 | <0.001 | <0.001 | 0.276 | <0.001 | <0.001 | 0.167 |
| SAT, cm^2^ | <0.001 | <0.001 | 0.706 | <0.001 | <0.001 | 0.953 | <0.001 | <0.001 | 0.905 |
| *Serum Lipids* | | | | | | | | | |
| TAG, mmol/L | <0.001 | <0.001 | 0.765 | <0.001 | <0.001 | 0.011 | <0.001 | <0.001 | 0.019 |
| T-C, mmol/L | <0.001 | 0.133 | 0.766 | <0.001 | 0.084 | 0.626 | <0.001 | 0.058 | 0.722 |
| HDL-C, mmol/L | <0.001 | <0.001 | 0.195 | <0.001 | <0.001 | 0.700 | <0.001 | <0.001 | 0.276 |
| LDL-C, mmol/L | 0.056 | <0.001 | 0.726 | 0.049 | <0.001 | 0.932 | 0.062 | <0.001 | 0.736 |
| T-C:HDL-C ratio | 0.008 | <0.001 | 0.309 | 0.005 | <0.001 | 0.974 | 0.004 | <0.001 | 0.212 |

**Table S2.** *p*-values for interaction between dietary fat intake (%E) and *IL-6* polymorphisms (genotype) on body composition in black and white adequate reporter women, adjusted for age.

| **Dietary variable** | **Black** | | | | | | | | **White** | | | | | | | |
| --- | --- | --- | --- | --- | --- | --- | --- | --- | --- | --- | --- | --- | --- | --- | --- | --- |
|  | **Weight, kg** | **BMI, kg/m^2^** | **Body fat, %** | **Fat Mass** | **Waist, cm** | **WHR** | **VAT, cm^2^** | **SAT, cm^2^** | **Weight, kg** | **BMI, kg/m^2^** | **Body fat, %** | **Fat Mass** | **Waist, cm** | **WHR** | **VAT, cm^2^** | **SAT, cm^2^** |
|  | ***IL-6* −174 G>C *n* = 144** | | | | | | | | ***IL-6* −174 G>C *n* = 122** | | | | | | | |
| Fat, %E | 0.271 | 0.169 | 0.184 | 0.186 | 0.412 | 0.467 | 0.265 | 0.738 | 0.687 | 0.634 | 0.419 | 0.562 | 0.796 | 0.495 | 0.483 | 0.390 |
| SFA, %E | 0.199 | 0.097 | 0.149 | 0.160 | 0.481 | 0.915 | 0.237 | 0.515 | 0.852 | 0.803 | 0.888 | 0.816 | 0.853 | 0.708 | 0.500 | 0.282 |
| MUFA, %E | 0.199 | 0.140 | 0.124 | 0.135 | 0.376 | 0.616 | 0.255 | 0.417 | 0.810 | 0.659 | 0.636 | 0.829 | 0.749 | 0.329 | 0.460 | 0.444 |
| PUFA, %E | 0.198 | 0.147 | 0.087 | 0.148 | 0.387 | 0.581 | 0.296 | 0.099 | 0.469 | 0.546 | 0.408 | 0.685 | 0.654 | 0.637 | 0.958 | 0.913 |
| P:S ratio | 0.086 | 0.038 | 0.031 | 0.051 | 0.224 | 0.926 | 0.147 | 0.148 | 0.208 | 0.203 | 0.548 | 0.426 | 0.237 | 0.896 | 0.344 | 0.191 |
| *n*-3 PUFA, %E | 0.218 | 0.260 | 0.164 | 0.186 | 0.278 | 0.461 | 0.390 | 0.149 | 0.141 | 0.068 | 0.619 | 0.330 | 0.169 | 0.599 | 0.133 | 0.059 |
| *n*-6 PUFA, %E | 0.142 | 0.099 | 0.054 | 0.097 | 0.312 | 0.686 | 0.284 | 0.111 | 0.559 | 0.603 | 0.530 | 0.769 | 0.701 | 0.599 | 0.959 | 0.898 |
| *n*-6:*n*-3 PUFA ratio | 0.062 | 0.059 | 0.021 | 0.037 | 0.109 | 0.450 | 0.294 | 0.120 | 0.100 | 0.041 | 0.434 | 0.204 | 0.068 | 0.904 | 0.188 | 0.139 |
| ALA, %E | 0.071 | 0.031 | 0.025 | 0.035 | 0.157 | 0.667 | 0.208 | 0.159 | 0.259 | 0.143 | 0.895 | 0.466 | 0.264 | 0.283 | 0.127 | 0.139 |
| LA, %E | 0.130 | 0.087 | 0.047 | 0.087 | 0.295 | 0.720 | 0.268 | 0.113 | 0.557 | 0.600 | 0.533 | 0.770 | 0.698 | 0.597 | 0.959 | 0.895 |
| AA, %E | 0.385 | 0.515 | 0.323 | 0.341 | 0.416 | 0.378 | 0.644 | 0.417 | 0.371 | 0.237 | 0.151 | 0.210 | 0.530 | 0.708 | 0.915 | 0.752 |
| EPA, %E | 0.499 | 0.685 | 0.507 | 0.530 | 0.507 | 0.418 | 0.433 | 0.649 | 0.168 | 0.097 | 0.137 | 0.187 | 0.150 | 0.729 | 0.480 | 0.207 |
| DHA, %E | 0.589 | 0.852 | 0.620 | 0.640 | 0.547 | 0.415 | 0.273 | 0.881 | 0.118 | 0.088 | 0.152 | 0.161 | 0.109 | 0.595 | 0.347 | 0.174 |

**Table S2.** *Cont.*

| **Dietary variable** | **Black** | | | | | | | | **White** | | | | | | | |
| --- | --- | --- | --- | --- | --- | --- | --- | --- | --- | --- | --- | --- | --- | --- | --- | --- |
|  | **Weight, kg** | **BMI, kg/m^2^** | **Body Fat, %** | **Fat Mass** | **Waist, cm** | **WHR** | **VAT, cm^2^** | **SAT, cm^2^** | **Weight, kg** | **BMI, kg/m^2^** | **Body Fat, %** | **Fat Mass** | **Waist, cm** | **WHR** | **VAT, cm^2^** | **SAT, cm^2^** |
|  | ***IL-6* IVS3 +281 G>T *n* = 130** | | | | | | | | ***IL-6* IVS3 +281 G>T *n* = 121** | | | | | | | |
| Fat, %E | 0.011 | 0.009 | 0.137 | 0.005 | 0.015 | 0.563 | 0.033 | 0.037 | 0.231 | 0.510 | 0.602 | 0.320 | 0.310 | 0.540 | 0.265 | 0.760 |
| SFA, %E | 0.017 | 0.007 | 0.167 | 0.006 | 0.058 | 0.617 | 0.081 | 0.242 | 0.341 | 0.569 | 0.528 | 0.360 | 0.234 | 0.611 | 0.204 | 0.668 |
| MUFA, %E | 0.010 | 0.010 | 0.357 | 0.008 | 0.040 | 0.846 | 0.009 | 0.043 | 0.446 | 0.979 | 0.921 | 0.569 | 0.824 | 0.090 | 0.985 | 0.598 |
| PUFA, %E | 0.112 | 0.104 | 0.626 | 0.097 | 0.112 | 0.561 | 0.201 | 0.098 | 0.406 | 0.352 | 0.429 | 0.450 | 0.590 | 0.229 | 0.397 | 0.458 |
| P:S ratio | 0.410 | 0.607 | 0.787 | 0.590 | 0.411 | 0.838 | 0.572 | 0.407 | 0.808 | 0.489 | 0.697 | 0.820 | 0.899 | 0.185 | 0.814 | 0.488 |
| *n*-3 PUFA, %E | 0.816 | 0.749 | 0.607 | 0.654 | 1.000 | 0.399 | 0.019 | 0.467 | 0.163 | 0.138 | 0.092 | 0.034 | 0.186 | 0.192 | 0.043 | 0.051 |
| *n*-6 PUFA, %E | 0.147 | 0.156 | 0.654 | 0.140 | 0.156 | 0.457 | 0.226 | 0.240 | 0.508 | 0.417 | 0.409 | 0.426 | 0.730 | 0.147 | 0.549 | 0.511 |
| *n*-6:*n*-3 PUFA ratio | 0.291 | 0.400 | 0.381 | 0.323 | 0.233 | 0.376 | 0.061 | 0.127 | 0.199 | 0.166 | 0.090 | 0.054 | 0.324 | 0.709 | 0.102 | 0.084 |
| ALA, %E | 0.303 | 0.300 | 0.363 | 0.210 | 0.706 | 0.456 | 0.102 | 0.492 | 0.096 | 0.092 | 0.141 | 0.048 | 0.142 | 0.172 | 0.006 | 0.007 |
| LA, %E | 0.157 | 0.166 | 0.670 | 0.151 | 0.165 | 0.470 | 0.235 | 0.254 | 0.505 | 0.414 | 0.406 | 0.423 | 0.727 | 0.145 | 0.548 | 0.506 |
| AA, %E | 0.016 | 0.010 | 0.433 | 0.014 | 0.052 | 0.646 | 0.254 | 0.909 | 0.581 | 0.585 | 0.468 | 0.503 | 0.576 | 0.885 | 0.746 | 0.930 |
| EPA, %E | 0.535 | 0.446 | 0.664 | 0.318 | 0.915 | 0.415 | 0.057 | 0.599 | 0.448 | 0.273 | 0.090 | 0.074 | 0.484 | 0.852 | 0.488 | 0.514 |
| DHA, %E | 0.470 | 0.405 | 0.767 | 0.304 | 0.888 | 0.444 | 0.052 | 0.682 | 0.455 | 0.289 | 0.240 | 0.135 | 0.507 | 0.974 | 0.646 | 0.501 |

**Table S2.** *Cont.*

| **Dietary variable** | **Black** | | | | | | | | **White** | | | | | | | |
| --- | --- | --- | --- | --- | --- | --- | --- | --- | --- | --- | --- | --- | --- | --- | --- | --- |
|  | **Weight, kg** | **BMI, kg/m^2^** | **Body Fat, %** | **Fat Mass** | **Waist, cm** | **WHR** | **VAT, cm^2^** | **SAT, cm^2^** | **Weight, kg** | **BMI, kg/m^2^** | **Body Fat, %** | **Fat Mass** | **Waist, cm** | **WHR** | **VAT, cm^2^** | **SAT, cm^2^** |
|  | ***IL-6* IVS4 +869 A>G *n* = 130** | | | | | | | | ***IL-6* IVS4 +869 A>G *n* = 121** | | | | | | | |
| Fat, %E | 0.020 | 0.012 | 0.129 | 0.006 | 0.032 | 0.597 | 0.054 | 0.112 | 0.914 | 0.859 | 0.988 | 0.853 | 0.801 | 0.437 | 0.433 | 0.502 |
| SFA, %E | 0.060 | 0.031 | 0.249 | 0.024 | 0.148 | 0.592 | 0.253 | 0.715 | 0.936 | 0.911 | 0.832 | 0.930 | 0.983 | 0.683 | 0.739 | 0.632 |
| MUFA, %E | 0.052 | 0.046 | 0.629 | 0.038 | 0.158 | 0.538 | 0.025 | 0.257 | 0.958 | 0.816 | 0.981 | 0.893 | 0.790 | 0.317 | 0.466 | 0.553 |
| PUFA, %E | 0.020 | 0.014 | 0.616 | 0.013 | 0.041 | 0.575 | 0.110 | 0.051 | 0.956 | 0.848 | 0.686 | 0.962 | 0.983 | 0.477 | 0.881 | 0.860 |
| P:S ratio | 0.205 | 0.255 | 0.768 | 0.267 | 0.264 | 0.841 | 0.529 | 0.189 | 0.811 | 0.674 | 0.698 | 0.805 | 0.900 | 0.622 | 0.640 | 0.639 |
| *n*-3 PUFA, %E | 0.961 | 0.942 | 0.652 | 0.924 | 0.868 | 0.510 | 0.008 | 0.233 | 0.409 | 0.209 | 0.427 | 0.369 | 0.237 | 0.182 | 0.426 | 0.270 |
| *n*-6 PUFA, %E | 0.025 | 0.019 | 0.674 | 0.019 | 0.051 | 0.491 | 0.116 | 0.126 | 0.909 | 0.729 | 0.527 | 0.879 | 0.955 | 0.461 | 0.815 | 0.734 |
| *n*-6:*n*-3 PUFA ratio | 0.075 | 0.095 | 0.762 | 0.122 | 0.085 | 0.517 | 0.024 | 0.064 | 0.254 | 0.078 | 0.128 | 0.198 | 0.101 | 0.537 | 0.265 | 0.245 |
| ALA, %E | 0.746 | 0.805 | 0.636 | 0.613 | 0.844 | 0.521 | 0.028 | 0.248 | 0.270 | 0.098 | 0.456 | 0.259 | 0.099 | 0.070 | 0.101 | 0.110 |
| LA, %E | 0.027 | 0.021 | 0.679 | 0.021 | 0.055 | 0.501 | 0.124 | 0.134 | 0.905 | 0.724 | 0.520 | 0.872 | 0.953 | 0.461 | 0.812 | 0.731 |
| AA, %E | 0.012 | 0.007 | 0.214 | 0.008 | 0.067 | 0.794 | 0.359 | 0.882 | 0.265 | 0.235 | 0.205 | 0.305 | 0.495 | 0.816 | 0.781 | 0.703 |
| EPA, %E | 0.708 | 0.620 | 0.739 | 0.506 | 0.966 | 0.575 | 0.035 | 0.425 | 0.462 | 0.453 | 0.103 | 0.281 | 0.416 | 0.814 | 0.827 | 0.730 |
| DHA, %E | 0.621 | 0.549 | 0.947 | 0.449 | 0.937 | 0.636 | 0.035 | 0.548 | 0.455 | 0.401 | 0.237 | 0.334 | 0.353 | 0.698 | 0.556 | 0.627 |

**Table S3.** *p*-values for interaction between dietary fat intake (%E) and *IL-6* polymorphisms (allelic) on body composition in 144 black and 122 white adequate reporter women, adjusted for age.

| **Dietary variable** | **Black** | | | | | | | | **White** | | | | | | | |
| --- | --- | --- | --- | --- | --- | --- | --- | --- | --- | --- | --- | --- | --- | --- | --- | --- |
|  | **Weight, kg** | **BMI, kg/m^2^** | **Body Fat, %** | **Fat Mass** | **Waist, cm** | **WHR** | **VAT, cm^2^** | **SAT, cm^2^** | **Weight, kg** | **BMI, kg/m^2^** | **Body Fat, %** | **Fat Mass** | **Waist, cm** | **WHR** | **VAT, cm^2^** | **SAT, cm^2^** |
|  | ***IL-6* −174 G>C *n* = 144** | | | | | | | | ***IL-6* −174 G>C *n* = 122** | | | | | | | |
| Fat, %E | 0.271 | 0.169 | 0.184 | 0.186 | 0.412 | 0.467 | 0.265 | 0.738 | 0.450 | 0.376 | 0.593 | 0.343 | 0.468 | 0.299 | 0.386 | 0.183 |
| SFA, %E | 0.199 | 0.097 | 0.149 | 0.160 | 0.481 | 0.915 | 0.237 | 0.515 | 0.691 | 0.576 | 0.890 | 0.578 | 0.903 | 0.865 | 0.607 | 0.320 |
| MUFA, %E | 0.199 | 0.140 | 0.124 | 0.135 | 0.376 | 0.616 | 0.255 | 0.417 | 0.641 | 0.390 | 0.915 | 0.613 | 0.452 | 0.144 | 0.360 | 0.230 |
| PUFA, %E | 0.198 | 0.147 | 0.087 | 0.148 | 0.387 | 0.581 | 0.296 | 0.099 | 0.988 | 0.688 | 0.902 | 0.928 | 0.912 | 0.304 | 0.892 | 0.845 |
| P:S ratio | 0.086 | 0.038 | 0.031 | 0.051 | 0.224 | 0.926 | 0.147 | 0.148 | 0.649 | 0.365 | 0.889 | 0.590 | 0.624 | 0.589 | 0.448 | 0.324 |
| *n*-3 PUFA, %E | 0.218 | 0.260 | 0.164 | 0.186 | 0.278 | 0.461 | 0.390 | 0.149 | 0.053 | 0.027 | 0.421 | 0.151 | 0.061 | 0.322 | 0.120 | 0.060 |
| *n*-6 PUFA, %E | 0.142 | 0.099 | 0.054 | 0.097 | 0.312 | 0.686 | 0.284 | 0.111 | 0.926 | 0.627 | 0.950 | 0.960 | 0.887 | 0.263 | 0.927 | 0.751 |
| *n*-6:*n*-3 PUFA ratio | 0.062 | 0.059 | 0.021 | 0.037 | 0.109 | 0.450 | 0.294 | 0.120 | 0.085 | 0.028 | 0.379 | 0.150 | 0.054 | 0.759 | 0.101 | 0.070 |
| ALA, %E | 0.071 | 0.031 | 0.025 | 0.035 | 0.157 | 0.667 | 0.208 | 0.159 | 0.107 | 0.055 | 0.687 | 0.253 | 0.130 | 0.436 | 0.058 | 0.093 |
| LA, %E | 0.130 | 0.087 | 0.047 | 0.087 | 0.295 | 0.720 | 0.268 | 0.113 | 0.921 | 0.623 | 0.961 | 0.970 | 0.883 | 0.261 | 0.924 | 0.745 |
| AA, %E | 0.385 | 0.515 | 0.323 | 0.341 | 0.416 | 0.378 | 0.644 | 0.417 | 0.681 | 0.714 | 0.506 | 0.487 | 0.607 | 0.486 | 0.923 | 0.716 |
| EPA, %E | 0.499 | 0.685 | 0.507 | 0.530 | 0.507 | 0.418 | 0.433 | 0.649 | 0.069 | 0.040 | 0.118 | 0.102 | 0.101 | 0.586 | 0.434 | 0.109 |
| DHA, %E | 0.589 | 0.852 | 0.620 | 0.640 | 0.547 | 0.415 | 0.273 | 0.881 | 0.054 | 0.043 | 0.106 | 0.089 | 0.073 | 0.467 | 0.572 | 0.131 |

**Table S3.** *Cont.*

| **Dietary variable** | **Black** | | | | | | | | **White** | | | | | | | |
| --- | --- | --- | --- | --- | --- | --- | --- | --- | --- | --- | --- | --- | --- | --- | --- | --- |
|  | **Weight, kg** | **BMI, kg/m^2^** | **Body Fat, %** | **Fat Mass** | **Waist, cm** | **WHR** | **VAT, cm^2^** | **SAT, cm^2^** | **Weight, kg** | **BMI, kg/m^2^** | **Body Fat, %** | **Fat Mass** | **Waist, cm** | **WHR** | **VAT, cm^2^** | **SAT, cm^2^** |
|  | ***IL-6* IVS3 +281 G>T *n* = 135** | | | | | | | | ***IL-6* IVS3 +281 G>T *n* = 122** | | | | | | | |
| Fat, %E | 0.350 | 0.218 | 0.445 | 0.308 | 0.381 | 0.388 | 0.474 | 0.912 | 0.703 | 0.630 | 0.875 | 0.578 | 0.727 | 0.442 | 0.460 | 0.309 |
| SFA, %E | 0.485 | 0.506 | 0.118 | 0.662 | 0.871 | 0.718 | 0.652 | 0.934 | 0.761 | 0.745 | 0.816 | 0.852 | 0.906 | 0.899 | 0.504 | 0.352 |
| MUFA, %E | 0.418 | 0.303 | 0.476 | 0.408 | 0.494 | 0.349 | 0.526 | 0.733 | 0.831 | 0.558 | 0.860 | 0.661 | 0.543 | 0.157 | 0.373 | 0.289 |
| PUFA, %E | 0.288 | 0.162 | 0.705 | 0.154 | 0.177 | 0.151 | 0.384 | 0.815 | 0.756 | 0.572 | 0.447 | 0.881 | 0.840 | 0.237 | 0.840 | 0.794 |
| P:S ratio | 0.685 | 0.513 | 0.479 | 0.478 | 0.448 | 0.309 | 0.538 | 0.760 | 0.507 | 0.365 | 0.497 | 0.569 | 0.696 | 0.346 | 0.328 | 0.329 |
| *n*-3 PUFA, %E | 0.879 | 0.721 | 0.235 | 0.848 | 0.918 | 0.502 | 0.340 | 0.715 | 0.200 | 0.081 | 0.206 | 0.170 | 0.096 | 0.074 | 0.198 | 0.133 |
| *n*-6 PUFA, %E | 0.307 | 0.169 | 0.770 | 0.185 | 0.164 | 0.122 | 0.260 | 0.909 | 0.659 | 0.460 | 0.331 | 0.739 | 0.767 | 0.221 | 0.814 | 0.670 |
| *n*-6:*n*-3 PUFA ratio | 0.772 | 0.790 | 0.910 | 0.839 | 0.468 | 0.211 | 0.235 | 0.996 | 0.111 | 0.030 | 0.044 | 0.076 | 0.048 | 0.396 | 0.103 | 0.097 |
| ALA, %E | 0.362 | 0.482 | 0.180 | 0.317 | 0.510 | 0.557 | 0.209 | 0.413 | 0.107 | 0.032 | 0.241 | 0.110 | 0.038 | 0.037 | 0.029 | 0.037 |
| LA, %E | 0.303 | 0.168 | 0.770 | 0.185 | 0.163 | 0.127 | 0.260 | 0.926 | 0.653 | 0.455 | 0.326 | 0.730 | 0.762 | 0.221 | 0.811 | 0.665 |
| AA, %E | 0.314 | 0.270 | 0.250 | 0.188 | 0.829 | 0.466 | 0.180 | 0.826 | 0.386 | 0.417 | 0.360 | 0.342 | 0.423 | 0.580 | 0.659 | 0.521 |
| EPA, %E | 0.492 | 0.416 | 0.379 | 0.410 | 0.906 | 0.726 | 0.675 | 0.809 | 0.467 | 0.338 | 0.159 | 0.305 | 0.430 | 0.521 | 0.881 | 0.562 |
| DHA, %E | 0.434 | 0.375 | 0.616 | 0.298 | 0.839 | 0.648 | 0.510 | 0.777 | 0.537 | 0.404 | 0.292 | 0.417 | 0.453 | 0.400 | 0.837 | 0.721 |

**Table S3.** *Cont.*

| **Dietary variable** | **Black** | | | | | | | | **White** | | | | | | | |  |
| --- | --- | --- | --- | --- | --- | --- | --- | --- | --- | --- | --- | --- | --- | --- | --- | --- | --- |
|  | **Weight, kg** | **BMI, kg/m^2^** | **Body Fat, %** | **Fat Mass** | **Waist, cm** | **WHR** | **VAT, cm^2^** | **SAT, cm^2^** | **Weight, kg** | **BMI, kg/m^2^** | **Body Fat, %** | **Fat Mass** | **Waist, cm** | **WHR** | **VAT, cm^2^** | **SAT, cm^2^** | |
|  | ***IL-6* IVS4 +869 A>G *n* = 130** | | | | | | | | ***IL-6* IVS4 +869 A>G *n* = 121** | | | | | | | | |
| Fat, %E | 0.725 | 0.426 | 0.390 | 0.518 | 0.698 | 0.652 | 0.729 | 0.604 | 0.159 | 0.290 | 0.309 | 0.200 | 0.176 | 0.914 | 0.265 | 0.760 | |
| SFA, %E | 0.535 | 0.543 | 0.162 | 0.715 | 0.966 | 0.599 | 0.756 | 0.702 | 0.260 | 0.414 | 0.360 | 0.256 | 0.154 | 0.453 | 0.204 | 0.668 | |
| MUFA, %E | 0.693 | 0.542 | 0.413 | 0.623 | 0.916 | 0.917 | 0.938 | 0.776 | 0.286 | 0.607 | 0.517 | 0.343 | 0.508 | 0.252 | 0.985 | 0.598 | |
| PUFA, %E | 0.770 | 0.428 | 0.916 | 0.440 | 0.422 | 0.221 | 0.331 | 0.776 | 0.248 | 0.166 | 0.194 | 0.265 | 0.329 | 0.535 | 0.397 | 0.458 | |
| P:S ratio | 0.745 | 0.963 | 0.971 | 0.962 | 0.824 | 0.433 | 0.310 | 0.876 | 0.553 | 0.265 | 0.394 | 0.528 | 0.755 | 0.375 | 0.814 | 0.488 | |
| *n*-3 PUFA, %E | 0.984 | 0.919 | 0.510 | 0.910 | 0.757 | 0.371 | 0.208 | 0.790 | 0.319 | 0.328 | 0.258 | 0.112 | 0.417 | 0.423 | 0.043 | 0.051 | |
| *n*-6 PUFA, %E | 0.835 | 0.454 | 0.817 | 0.522 | 0.404 | 0.168 | 0.222 | 0.827 | 0.324 | 0.207 | 0.185 | 0.252 | 0.434 | 0.383 | 0.549 | 0.511 | |
| *n*-6:*n*-3 PUFA ratio | 0.833 | 0.981 | 0.525 | 0.820 | 0.664 | 0.229 | 0.157 | 0.866 | 0.148 | 0.111 | 0.052 | 0.034 | 0.231 | 0.869 | 0.102 | 0.084 | |
| ALA, %E | 0.234 | 0.268 | 0.141 | 0.186 | 0.279 | 0.239 | 0.127 | 0.203 | 0.179 | 0.199 | 0.294 | 0.112 | 0.285 | 0.323 | 0.006 | 0.007 | |
| LA, %E | 0.822 | 0.446 | 0.832 | 0.514 | 0.401 | 0.175 | 0.220 | 0.850 | 0.321 | 0.205 | 0.182 | 0.249 | 0.432 | 0.378 | 0.548 | 0.506 | |
| AA, %E | 0.518 | 0.488 | 0.320 | 0.295 | 0.928 | 0.340 | 0.116 | 0.856 | 0.840 | 0.927 | 0.840 | 0.803 | 0.957 | 0.502 | 0.746 | 0.930 | |
| EPA, %E | 0.496 | 0.454 | 0.783 | 0.390 | 0.964 | 0.607 | 0.537 | 0.994 | 0.632 | 0.474 | 0.209 | 0.163 | 0.742 | 0.864 | 0.488 | 0.514 | |
| DHA, %E | 0.459 | 0.427 | 0.932 | 0.283 | 0.884 | 0.587 | 0.391 | 0.959 | 0.553 | 0.393 | 0.344 | 0.197 | 0.645 | 0.876 | 0.646 | 0.501 | |

**Table S4.** *p*-values for interaction between dietary fat intake (%E) and *IL-6* polymorphisms (genotype) on body composition in 266 white and black adequate reporter women, adjusted for age and ethnicity.

| **Dietary variable** | **Weight, kg** | **BMI, kg/m^2^** | **Body Fat, %** | **Fat Mass** | **Waist, cm** | **WHR** | **VAT, cm^2^** | **SAT, cm^2^** |
| --- | --- | --- | --- | --- | --- | --- | --- | --- |
|  | ***IL-6* IVS3 +281 G>T *n* = 257** | | | | | | | |
| Fat, %E | 0.150 | 0.085 | 0.383 | 0.093 | 0.078 | 0.169 | 0.124 | 0.124 |
| SFA, %E | 0.736 | 0.578 | 0.515 | 0.630 | 0.622 | 0.554 | 0.409 | 0.699 |
| MUFA, %E | 0.448 | 0.188 | 0.889 | 0.308 | 0.300 | 0.146 | 0.065 | 0.270 |
| PUFA, %E | 0.416 | 0.359 | 0.557 | 0.354 | 0.289 | 0.130 | 0.780 | 0.268 |
| P:S ratio | 0.770 | 0.809 | 0.604 | 0.718 | 0.672 | 0.383 | 0.976 | 0.586 |
| *n*-3 PUFA, %E | 0.433 | 0.222 | 0.692 | 0.299 | 0.384 | 0.595 | 0.212 | 0.420 |
| *n*-6 PUFA, %E | 0.459 | 0.444 | 0.559 | 0.411 | 0.349 | 0.120 | 0.773 | 0.336 |
| *n*-6:*n*-3 PUFA ratio | 0.378 | 0.376 | 0.421 | 0.276 | 0.434 | 0.649 | 0.457 | 0.154 |
| ALA, %E | 0.645 | 0.465 | 0.707 | 0.392 | 0.567 | 0.659 | 0.244 | 0.827 |
| LA, %E | 0.475 | 0.463 | 0.563 | 0.430 | 0.361 | 0.126 | 0.781 | 0.351 |
| AA, %E | 0.385 | 0.352 | 0.896 | 0.366 | 0.551 | 0.922 | 0.735 | 0.942 |
| EPA, %E | 0.304 | 0.182 | 0.377 | 0.219 | 0.374 | 0.614 | 0.144 | 0.480 |
| DHA, %E | 0.340 | 0.211 | 0.451 | 0.218 | 0.375 | 0.641 | 0.094 | 0.476 |
| ***IL-6* IVS4 +869 A>G *n* = 251** | | | | | | | | |
| Fat, %E | 0.009 | 0.029 | 0.179 | 0.011 | 0.018 | 0.861 | 0.069 | 0.042 |
| SFA, %E | 0.014 | 0.009 | 0.378 | 0.015 | 0.049 | 0.759 | 0.050 | 0.255 |
| MUFA, %E | 0.057 | 0.113 | 0.747 | 0.096 | 0.260 | 0.510 | 0.121 | 0.131 |
| PUFA, %E | 0.157 | 0.272 | 0.310 | 0.187 | 0.275 | 0.559 | 0.380 | 0.135 |
| P:S ratio | 0.461 | 0.523 | 0.559 | 0.514 | 0.747 | 0.613 | 0.899 | 0.450 |
| *n*-3 PUFA, %E | 0.645 | 0.678 | 0.606 | 0.393 | 0.620 | 0.106 | 0.010 | 0.139 |
| *n*-6 PUFA, %E | 0.201 | 0.350 | 0.288 | 0.203 | 0.376 | 0.423 | 0.420 | 0.194 |
| *n*-6:*n*-3 PUFA ratio | 0.096 | 0.167 | 0.053 | 0.035 | 0.171 | 0.444 | 0.041 | 0.030 |
| ALA, %E | 0.774 | 0.776 | 0.845 | 0.722 | 0.894 | 0.296 | 0.049 | 0.420 |
| LA, %E | 0.209 | 0.364 | 0.289 | 0.211 | 0.385 | 0.444 | 0.431 | 0.201 |
| AA, %E | 0.208 | 0.184 | 0.688 | 0.306 | 0.472 | 0.415 | 0.795 | 0.708 |
| EPA, %E | 0.807 | 0.688 | 0.694 | 0.483 | 0.893 | 0.137 | 0.073 | 0.502 |
| DHA, %E | 0.656 | 0.561 | 0.707 | 0.374 | 0.941 | 0.198 | 0.133 | 0.578 |

**Table S5.** *p*-values for interaction between dietary fat intake (%E) and *IL-6* polymorphisms (allelic) on body composition in 266 white and black adequate reporter women, adjusted for age and ethnicity.

| **Dietary variable** | **Weight, kg** | **BMI, kg/m^2^** | **Body Fat, %** | **Fat Mass** | **Waist, cm** | **WHR** | **VAT, cm^2^** | **SAT, cm^2^** |
| --- | --- | --- | --- | --- | --- | --- | --- | --- |
|  | ***IL-6* IVS3 +281 G>T *n* = 257** | | | | | | | |
| Fat, %E | 0.487 | 0.266 | 0.670 | 0.454 | 0.466 | 0.209 | 0.315 | 0.523 |
| SFA, %E | 0.763 | 0.692 | 0.279 | 0.746 | 0.854 | 0.933 | 0.520 | 0.640 |
| MUFA, %E | 0.477 | 0.222 | 0.851 | 0.350 | 0.313 | 0.064 | 0.198 | 0.265 |
| PUFA, %E | 0.579 | 0.428 | 0.958 | 0.576 | 0.368 | 0.070 | 0.620 | 0.825 |
| P:S ratio | 0.958 | 0.864 | 0.762 | 0.939 | 0.635 | 0.208 | 0.923 | 0.763 |
| *n*-3 PUFA, %E | 0.229 | 0.098 | 0.549 | 0.147 | 0.211 | 0.382 | 0.217 | 0.350 |
| *n*-6 PUFA, %E | 0.678 | 0.530 | 0.886 | 0.731 | 0.403 | 0.061 | 0.598 | 0.893 |
| *n*-6:*n*-3 PUFA ratio | 0.480 | 0.355 | 0.312 | 0.265 | 0.616 | 0.548 | 0.633 | 0.507 |
| ALA, %E | 0.637 | 0.463 | 0.974 | 0.578 | 0.430 | 0.396 | 0.103 | 0.516 |
| LA, %E | 0.678 | 0.535 | 0.890 | 0.746 | 0.410 | 0.067 | 0.595 | 0.880 |
| AA, %E | 0.263 | 0.234 | 0.781 | 0.247 | 0.502 | 0.996 | 0.940 | 0.798 |
| EPA, %E | 0.191 | 0.084 | 0.248 | 0.108 | 0.278 | 0.481 | 0.473 | 0.531 |
| DHA, %E | 0.210 | 0.098 | 0.315 | 0.111 | 0.284 | 0.497 | 0.923 | 0.724 |
| ***IL-6* IVS4 +869 A>G *n* = 251** | | | | | | | | |
| Fat, %E | 0.443 | 0.896 | 0.204 | 0.571 | 0.503 | 0.634 | 0.734 | 0.459 |
| SFA, %E | 0.715 | 0.935 | 0.123 | 0.632 | 0.362 | 0.380 | 0.185 | 0.537 |
| MUFA, %E | 0.685 | 0.860 | 0.394 | 0.766 | 0.729 | 0.419 | 0.890 | 0.969 |
| PUFA, %E | 0.428 | 0.660 | 0.378 | 0.534 | 0.773 | 0.173 | 0.899 | 0.491 |
| P:S ratio | 0.373 | 0.417 | 0.607 | 0.422 | 0.760 | 0.217 | 0.629 | 0.557 |
| *n*-3 PUFA, %E | 0.548 | 0.560 | 0.735 | 0.312 | 0.907 | 0.551 | 0.287 | 0.437 |
| *n*-6 PUFA, %E | 0.432 | 0.663 | 0.331 | 0.467 | 0.846 | 0.114 | 0.700 | 0.535 |
| *n*-6:*n*-3 PUFA ratio | 0.241 | 0.336 | 0.133 | 0.123 | 0.659 | 0.172 | 0.804 | 0.322 |
| ALA, %E | 0.863 | 0.992 | 0.829 | 0.792 | 0.922 | 0.566 | 0.164 | 0.568 |
| LA, %E | 0.432 | 0.659 | 0.338 | 0.461 | 0.837 | 0.120 | 0.697 | 0.544 |
| AA, %E | 0.917 | 0.916 | 0.507 | 0.805 | 0.629 | 0.305 | 0.602 | 0.657 |
| EPA, %E | 0.540 | 0.403 | 0.504 | 0.242 | 0.958 | 0.663 | 0.488 | 0.850 |
| DHA, %E | 0.440 | 0.329 | 0.485 | 0.178 | 0.831 | 0.574 | 0.728 | 0.833 |

**Table S6.** *p*-values for 3-way interaction between ethnic group, dietary fat intake (%E) and *IL-6* polymorphisms (genotype) on body composition in 266 white and black adequate reporter women, adjusted for age.

| **Dietary variable** | **Weight, kg** | **BMI, kg/m^2^** | **Body Fat, %** | **Fat Mass** | **Waist, cm** | **WHR** | **VAT, cm^2^** | **SAT, cm^2^** |
| --- | --- | --- | --- | --- | --- | --- | --- | --- |
|  | ***IL-6* IVS3 +281 G>T *n* = 257** | | | | | | | |
| Fat, %E | 0.171 | 0.114 | 0.512 | 0.118 | 0.259 | 0.957 | 0.962 | 0.429 |
| SFA, %E | 0.112 | 0.045 | 0.424 | 0.054 | 0.241 | 0.856 | 0.605 | 0.872 |
| MUFA, %E | 0.144 | 0.162 | 0.815 | 0.154 | 0.418 | 0.980 | 0.536 | 0.504 |
| PUFA, %E | 0.057 | 0.024 | 0.725 | 0.075 | 0.077 | 0.975 | 0.566 | 0.228 |
| P:S ratio | 0.267 | 0.231 | 0.758 | 0.400 | 0.249 | 0.990 | 0.570 | 0.348 |
| *n*-3 PUFA, %E | 0.692 | 0.622 | 0.348 | 0.588 | 0.455 | 0.156 | 0.063 | 0.134 |
| *n*-6 PUFA, %E | 0.065 | 0.027 | 0.680 | 0.082 | 0.085 | 0.961 | 0.512 | 0.376 |
| *n*-6:*n*-3 PUFA ratio | 0.055 | 0.023 | 0.500 | 0.081 | 0.015 | 0.224 | 0.077 | 0.151 |
| ALA, %E | 0.384 | 0.313 | 0.604 | 0.410 | 0.306 | 0.141 | 0.022 | 0.052 |
| LA, %E | 0.069 | 0.029 | 0.679 | 0.086 | 0.089 | 0.963 | 0.518 | 0.387 |
| AA, %E | 0.010 | 0.005 | 0.045 | 0.016 | 0.054 | 0.692 | 0.668 | 0.576 |
| EPA, %E | 0.643 | 0.645 | 0.087 | 0.339 | 0.639 | 0.515 | 0.445 | 0.484 |
| DHA, %E | 0.554 | 0.507 | 0.282 | 0.400 | 0.570 | 0.510 | 0.619 | 0.617 |
| ***IL-6* IVS4 +869 A>G *n* = 251** | | | | | | | | |
| Fat, %E | 0.761 | 0.518 | 0.476 | 0.718 | 0.591 | 0.358 | 0.683 | 0.137 |
| SFA, %E | 0.834 | 0.479 | 0.352 | 0.588 | 0.725 | 0.583 | 0.946 | 0.374 |
| MUFA, %E | 0.706 | 0.359 | 0.488 | 0.665 | 0.404 | 0.157 | 0.534 | 0.105 |
| PUFA, %E | 0.915 | 0.865 | 0.889 | 0.923 | 0.887 | 0.605 | 0.523 | 0.389 |
| P:S ratio | 0.477 | 0.884 | 0.886 | 0.671 | 0.409 | 0.497 | 0.639 | 0.614 |
| *n*-3 PUFA, %E | 0.231 | 0.204 | 0.099 | 0.062 | 0.275 | 0.368 | 0.179 | 0.231 |
| *n*-6 PUFA, %E | 0.833 | 0.867 | 0.869 | 0.880 | 0.872 | 0.582 | 0.529 | 0.590 |
| *n*-6:*n*-3 PUFA ratio | 0.974 | 0.827 | 0.635 | 0.588 | 0.972 | 0.807 | 0.280 | 0.992 |
| ALA, %E | 0.027 | 0.027 | 0.046 | 0.010 | 0.093 | 0.223 | 0.007 | 0.011 |
| LA, %E | 0.850 | 0.847 | 0.856 | 0.863 | 0.885 | 0.571 | 0.520 | 0.609 |
| AA, %E | 0.134 | 0.101 | 0.167 | 0.183 | 0.080 | 0.494 | 0.963 | 0.601 |
| EPA, %E | 0.626 | 0.452 | 0.127 | 0.175 | 0.522 | 0.916 | 0.905 | 0.865 |
| DHA, %E | 0.635 | 0.472 | 0.414 | 0.356 | 0.568 | 0.909 | 0.862 | 0.819 |

**Table S7.** *p*-values for 3-way interaction between ethnic group, dietary fat intake (%E) and *IL-6* polymorphisms (allelic) on body composition in 266 white and black adequate reporter women, adjusted for age.

| **Dietary variable** | **Weight, kg** | **BMI, kg/m^2^** | **Body Fat, %** | **Fat Mass** | **Waist, cm** | **WHR** | **VAT, cm^2^** | **SAT, cm^2^** |
| --- | --- | --- | --- | --- | --- | --- | --- | --- |
|  | ***IL-6* IVS3 +281 G>T *n* = 257** | | | | | | | |
| Fat, %E | 0.749 | 0.609 | 0.710 | 0.833 | 0.735 | 0.909 | 0.769 | 0.434 |
| SFA, %E | 0.717 | 0.716 | 0.283 | 0.835 | 0.862 | 0.777 | 0.912 | 0.639 |
| MUFA, %E | 0.667 | 0.688 | 0.718 | 0.810 | 0.923 | 0.910 | 0.744 | 0.696 |
| PUFA, %E | 0.336 | 0.157 | 0.402 | 0.293 | 0.264 | 0.718 | 0.577 | 0.958 |
| P:S ratio | 0.430 | 0.259 | 0.326 | 0.351 | 0.393 | 0.818 | 0.292 | 0.608 |
| *n*-3 PUFA, %E | 0.384 | 0.327 | 0.086 | 0.347 | 0.196 | 0.091 | 0.196 | 0.179 |
| *n*-6 PUFA, %E | 0.301 | 0.128 | 0.344 | 0.256 | 0.223 | 0.671 | 0.473 | 0.794 |
| *n*-6:*n*-3 PUFA | 0.151 | 0.078 | 0.097 | 0.124 | 0.049 | 0.135 | 0.056 | 0.205 |
| ALA, %E | 0.076 | 0.055 | 0.080 | 0.066 | 0.062 | 0.086 | 0.024 | 0.051 |
| LA, %E | 0.295 | 0.126 | 0.340 | 0.251 | 0.221 | 0.677 | 0.469 | 0.780 |
| AA, %E | 0.999 | 0.851 | 0.146 | 0.920 | 0.642 | 0.346 | 0.379 | 0.472 |
| EPA, %E | 0.924 | 0.916 | 0.111 | 0.800 | 0.604 | 0.470 | 0.946 | 0.509 |
| DHA, %E | 0.950 | 0.966 | 0.280 | 0.945 | 0.665 | 0.346 | 0.933 | 0.579 |
| ***IL-6* IVS4 +869 A>G *n* = 251** | | | | | | | | |
| Fat, %E | 0.168 | 0.206 | 0.636 | 0.147 | 0.201 | 0.922 | 0.164 | 0.924 |
| SFA, %E | 0.227 | 0.338 | 0.757 | 0.289 | 0.344 | 0.999 | 0.319 | 0.935 |
| MUFA, %E | 0.276 | 0.463 | 0.895 | 0.287 | 0.594 | 0.383 | 0.856 | 0.498 |
| PUFA, %E | 0.251 | 0.115 | 0.271 | 0.163 | 0.206 | 0.778 | 0.207 | 0.654 |
| P:S ratio | 0.752 | 0.362 | 0.448 | 0.566 | 0.692 | 0.890 | 0.528 | 0.627 |
| *n*-3 PUFA, %E | 0.391 | 0.446 | 0.188 | 0.176 | 0.361 | 0.210 | 0.027 | 0.071 |
| *n*-6 PUFA, %E | 0.339 | 0.145 | 0.297 | 0.176 | 0.256 | 0.827 | 0.274 | 0.670 |
| *n*-6:*n*-3 PUFA ratio | 0.268 | 0.199 | 0.188 | 0.091 | 0.216 | 0.518 | 0.032 | 0.183 |
| ALA, %E | 0.069 | 0.088 | 0.081 | 0.036 | 0.123 | 0.123 | 0.002 | 0.004 |
| LA, %E | 0.332 | 0.142 | 0.290 | 0.173 | 0.254 | 0.840 | 0.273 | 0.655 |
| AA, %E | 0.874 | 0.777 | 0.431 | 0.742 | 0.847 | 0.810 | 0.911 | 0.703 |
| EPA, %E | 0.982 | 0.896 | 0.245 | 0.464 | 0.770 | 0.772 | 0.489 | 0.553 |
| DHA, %E | 0.925 | 0.821 | 0.485 | 0.598 | 0.718 | 0.720 | 0.570 | 0.551 |

**Table S8.** *p*-values for interaction between dietary fat intake (%E) and *IL-6* polymorphisms (genotypes) on serum lipids in 144 black and 122 white adequate reporter women, adjusted for age and fat mass.

| **Dietary variable** | **Black** | | | | | **White** | | | | |
| --- | --- | --- | --- | --- | --- | --- | --- | --- | --- | --- |
|  | **TAG, mmol/L** | **T-C, mmol/L** | **HDL-C, mmol/L** | **LDL-C, mmol/L** | **T-C:HDL-C Ratio** | **TAG, mmol/L** | **T-C, mmol/L** | **HDL-C, mmol/L** | **LDL-C, mmol/L** | **T-C:HDL-C Ratio** |
|  | **IL-6 174 G>C, *n* = 144** | | | | | **IL-6 174 G>C, *n* = 122** | | | | |
| Fat, %E | 0.217 | 0.291 | 0.540 | 0.723 | 0.828 | 0.214 | 0.123 | 0.562 | 0.186 | 0.560 |
| SFA, %E | 0.129 | 0.056 | 0.416 | 0.261 | 0.851 | 0.346 | 0.064 | 0.405 | 0.115 | 0.574 |
| MUFA, %E | 0.054 | 0.469 | 0.714 | 0.564 | 0.362 | 0.105 | 0.107 | 0.681 | 0.142 | 0.421 |
| PUFA, %E | 0.423 | 0.680 | 0.359 | 0.706 | 0.295 | 0.425 | 0.701 | 0.834 | 0.607 | 0.621 |
| P:S ratio | 0.639 | 0.730 | 0.408 | 0.584 | 0.237 | 0.358 | 0.254 | 0.889 | 0.241 | 0.468 |
| *n*-3 PUFA, %E | 0.467 | 0.903 | 0.059 | 0.563 | 0.088 | 0.101 | 0.321 | 0.970 | 0.317 | 0.465 |
| *n*-6 PUFA, %E | 0.409 | 0.644 | 0.517 | 0.713 | 0.387 | 0.306 | 0.538 | 0.866 | 0.462 | 0.529 |
| *n*-6:*n*-3 PUFA ratio | 0.544 | 0.442 | 0.143 | 0.853 | 0.615 | 0.066 | 0.318 | 0.791 | 0.248 | 0.307 |
| ALA, %E | 0.846 | 0.957 | 0.071 | 0.856 | 0.157 | 0.141 | 0.117 | 0.799 | 0.123 | 0.351 |
| LA, %E | 0.406 | 0.644 | 0.523 | 0.715 | 0.390 | 0.288 | 0.497 | 0.892 | 0.432 | 0.520 |
| AA, %E | 0.919 | 0.879 | 0.404 | 0.346 | 0.334 | 0.153 | 0.571 | 0.668 | 0.442 | 0.388 |
| EPA, %E | 0.083 | 0.468 | 0.124 | 0.123 | 0.021 | 0.178 | 0.595 | 0.994 | 0.598 | 0.671 |
| DHA, %E | 0.122 | 0.547 | 0.060 | 0.159 | 0.014 | 0.244 | 0.823 | 0.794 | 0.752 | 0.660 |

**Table S8.** *Cont.*

| **Dietary variable** | **Black** | | | | | **White** | | | | |
| --- | --- | --- | --- | --- | --- | --- | --- | --- | --- | --- |
|  | **TAG, mmol/L** | **T-C, mmol/L** | **HDL-C, mmol/L** | **LDL-C, mmol/L** | **T-C:HDL-C Ratio** | **TAG, mmol/L** | **T-C, mmol/L** | **HDL-C, mmol/L** | **LDL-C, mmol/L** | **T-C:HDL-C Ratio** |
|  | **IVS3 +281 G>T *n* = 135** | | | | | **IVS3 +281 G>T *n* = 122** | | | | |
| Fat, %E | 0.777 | 0.667 | 0.514 | 0.593 | 0.754 | 0.090 | 0.107 | 0.966 | 0.051 | 0.187 |
| SFA, %E | 0.339 | 0.624 | 0.590 | 0.536 | 0.389 | 0.135 | 0.519 | 0.902 | 0.346 | 0.713 |
| MUFA, %E | 0.456 | 0.907 | 0.278 | 0.978 | 0.346 | 0.148 | 0.327 | 0.934 | 0.155 | 0.336 |
| PUFA, %E | 0.165 | 0.216 | 0.561 | 0.356 | 0.746 | 0.171 | 0.114 | 0.789 | 0.060 | 0.091 |
| P:S ratio | 0.144 | 0.181 | 0.825 | 0.257 | 0.465 | 0.758 | 0.192 | 0.740 | 0.184 | 0.192 |
| *n*-3 PUFA, %E | 0.874 | 0.647 | 0.554 | 0.393 | 0.380 | 0.988 | 0.831 | 0.308 | 0.898 | 0.532 |
| *n*-6 PUFA, %E | 0.112 | 0.176 | 0.424 | 0.401 | 0.845 | 0.211 | 0.148 | 0.821 | 0.095 | 0.126 |
| *n*-6:*n*-3 PUFA ratio | 0.577 | 0.601 | 0.216 | 0.816 | 0.425 | 0.419 | 0.190 | 0.372 | 0.147 | 0.186 |
| ALA, %E | 0.835 | 0.501 | 0.631 | 0.741 | 0.981 | 0.607 | 0.979 | 0.455 | 0.968 | 0.571 |
| LA, %E | 0.114 | 0.173 | 0.418 | 0.396 | 0.846 | 0.211 | 0.146 | 0.833 | 0.095 | 0.130 |
| AA, %E | 0.405 | 0.619 | 0.303 | 0.742 | 0.541 | 0.833 | 0.940 | 0.226 | 0.959 | 0.365 |
| EPA, %E | 0.561 | 0.233 | 0.065 | 0.752 | 0.379 | 0.825 | 0.824 | 0.506 | 0.862 | 0.681 |
| DHA, %E | 0.470 | 0.143 | 0.029 | 0.510 | 0.318 | 0.673 | 0.707 | 0.537 | 0.798 | 0.857 |

**Table S8.** *Cont.*

| **Dietary variable** | **Black** | | | | | **White** | | | | |
| --- | --- | --- | --- | --- | --- | --- | --- | --- | --- | --- |
|  | **TAG, mmol/L** | **T-C, mmol/L** | **HDL-C, mmol/L** | **LDL-C, mmol/L** | **T-C:HDL-C Ratio** | **TAG, mmol/L** | **T-C, mmol/L** | **HDL-C, mmol/L** | **LDL-C, mmol/L** | **T-C:HDL-C Ratio** |
|  | ***IL-6* IVS4 +869 A>G *n* = 130** | | | | | ***IL-6* IVS4 +869 A>G *n* = 121** | | | | |
| Fat, %E | 0.496 | 0.525 | 0.771 | 0.821 | 0.929 | 0.401 | 0.057 | 0.759 | 0.010 | 0.056 |
| SFA, %E | 0.480 | 0.188 | 0.284 | 0.468 | 0.821 | 0.080 | 0.702 | 0.980 | 0.520 | 0.784 |
| MUFA, %E | 0.464 | 0.355 | 0.341 | 0.444 | 0.360 | 0.665 | 0.192 | 0.597 | 0.040 | 0.082 |
| PUFA, %E | 0.261 | 0.757 | 0.974 | 0.917 | 0.952 | 0.513 | 0.109 | 0.522 | 0.023 | 0.034 |
| P:S ratio | 0.659 | 0.674 | 0.436 | 0.788 | 0.727 | 0.821 | 0.172 | 0.567 | 0.069 | 0.060 |
| *n*-3 PUFA, %E | 0.223 | 0.397 | 0.114 | 0.184 | 0.015 | 0.791 | 0.853 | 0.323 | 0.948 | 0.552 |
| *n*-6 PUFA, %E | 0.287 | 0.757 | 0.920 | 0.959 | 0.990 | 0.592 | 0.119 | 0.544 | 0.033 | 0.041 |
| *n*-6:*n*-3 PUFA ratio | 0.800 | 0.901 | 0.140 | 0.464 | 0.111 | 0.672 | 0.202 | 0.738 | 0.136 | 0.383 |
| ALA, %E | 0.239 | 0.764 | 0.059 | 0.274 | 0.029 | 0.888 | 0.813 | 0.616 | 0.674 | 0.375 |
| LA, %E | 0.284 | 0.756 | 0.919 | 0.958 | 0.991 | 0.578 | 0.118 | 0.550 | 0.033 | 0.043 |
| AA, %E | 0.913 | 0.325 | 0.780 | 0.230 | 0.776 | 0.991 | 0.918 | 0.333 | 0.970 | 0.488 |
| EPA, %E | 0.304 | 0.216 | 0.432 | 0.150 | 0.084 | 0.636 | 0.723 | 0.405 | 0.871 | 0.736 |
| DHA, %E | 0.169 | 0.122 | 0.301 | 0.075 | 0.055 | 0.493 | 0.566 | 0.371 | 0.762 | 0.826 |

**Table S9.** *p*-values for interaction between dietary fat intake (%E) and *IL-6* polymorphisms (allelic) on serum lipids in 144 black and 122 white adequate reporter women, adjusted for age and fat mass.

| **Dietary variable** | **Black** | | | | | **White** | | | | |
| --- | --- | --- | --- | --- | --- | --- | --- | --- | --- | --- |
|  | **TAG, mmol/L** | **T-C, mmol/L** | **HDL-C, mmol/L** | **LDL-C, mmol/L** | **T-C:HDL-C Ratio** | **TAG, mmol/L** | **T-C, mmol/L** | **HDL-C, mmol/L** | **LDL-C, mmol/L** | **T-C:HDL-C Ratio** |
|  | **IL-6 174 G>C, *n* = 144** | | | | | **IL-6 174 G>C, *n* = 122** | | | | |
| Fat, %E | 0.214 | 0.123 | 0.562 | 0.186 | 0.560 | 0.098 | 0.119 | 0.313 | 0.552 | 0.867 |
| SFA, %E | 0.346 | 0.064 | 0.405 | 0.115 | 0.574 | 0.131 | 0.017 | 0.198 | 0.094 | 0.623 |
| MUFA, %E | 0.105 | 0.107 | 0.681 | 0.142 | 0.421 | 0.015 | 0.239 | 0.535 | 0.321 | 0.154 |
| PUFA, %E | 0.425 | 0.701 | 0.834 | 0.607 | 0.621 | 0.520 | 0.564 | 0.385 | 0.983 | 0.687 |
| P:S ratio | 0.358 | 0.254 | 0.889 | 0.241 | 0.468 | 0.766 | 0.574 | 0.853 | 0.524 | 0.825 |
| *n*-3 PUFA, %E | 0.101 | 0.321 | 0.970 | 0.317 | 0.465 | 0.237 | 0.775 | 0.047 | 0.991 | 0.100 |
| *n*-6 PUFA, %E | 0.306 | 0.538 | 0.866 | 0.462 | 0.529 | 0.560 | 0.576 | 0.486 | 0.895 | 0.798 |
| *n*-6:*n*-3 PUFA ratio | 0.066 | 0.318 | 0.791 | 0.248 | 0.307 | 0.680 | 0.318 | 0.084 | 0.636 | 0.365 |
| ALA, %E | 0.141 | 0.117 | 0.799 | 0.123 | 0.351 | 0.628 | 0.768 | 0.022 | 0.568 | 0.056 |
| LA, %E | 0.288 | 0.497 | 0.892 | 0.432 | 0.520 | 0.562 | 0.580 | 0.488 | 0.899 | 0.797 |
| AA, %E | 0.153 | 0.571 | 0.668 | 0.442 | 0.388 | 0.866 | 0.694 | 0.850 | 0.600 | 0.910 |
| EPA, %E | 0.178 | 0.595 | 0.994 | 0.598 | 0.671 | 0.044 | 0.480 | 0.707 | 0.948 | 0.386 |
| DHA, %E | 0.244 | 0.823 | 0.794 | 0.752 | 0.660 | 0.068 | 0.374 | 0.465 | 0.432 | 0.184 |

**Table S9.** *Cont.*

| **Dietary variable** | **Black** | | | | | **White** | | | | |
| --- | --- | --- | --- | --- | --- | --- | --- | --- | --- | --- |
|  | **TAG, mmol/L** | **T-C, mmol/L** | **HDL-C, mmol/L** | **LDL-C, mmol/L** | **T-C:HDL-C Ratio** | **TAG, mmol/L** | **T-C, mmol/L** | **HDL-C, mmol/L** | **LDL-C, mmol/L** | **T-C:HDL-C Ratio** |
|  | **IVS3 +281 G>T *n* = 135** | | | | | **IVS3 +281 G>T *n* = 122** | | | | |
| Fat, %E | 0.478 | 0.173 | 0.849 | 0.725 | 0.516 | 0.300 | 0.714 | 0.419 | 0.841 | 0.650 |
| SFA, %E | 0.714 | 0.070 | 0.469 | 0.847 | 0.266 | 0.194 | 0.671 | 0.129 | 0.421 | 0.234 |
| MUFA, %E | 0.539 | 0.760 | 0.288 | 0.755 | 0.126 | 0.501 | 0.284 | 0.426 | 0.348 | 0.284 |
| PUFA, %E | 0.610 | 0.209 | 0.853 | 0.902 | 0.944 | 0.976 | 0.620 | 0.804 | 0.986 | 0.738 |
| P:S ratio | 0.771 | 0.755 | 0.872 | 0.838 | 0.837 | 0.377 | 0.962 | 0.391 | 0.446 | 0.505 |
| *n*-3 PUFA, %E | 0.124 | 0.604 | 0.875 | 0.174 | 0.515 | 0.416 | 0.191 | 0.407 | 0.091 | 0.246 |
| *n*-6 PUFA, %E | 0.699 | 0.274 | 0.947 | 0.834 | 0.873 | 0.948 | 0.544 | 0.741 | 0.890 | 0.806 |
| *n*-6:*n*-3 PUFA ratio | 0.232 | 0.282 | 0.644 | 0.304 | 0.423 | 0.419 | 0.556 | 0.655 | 0.303 | 0.501 |
| ALA, %E | 0.689 | 0.935 | 0.468 | 0.279 | 0.250 | 0.829 | 0.453 | 0.565 | **0.018** | 0.104 |
| LA, %E | 0.690 | 0.267 | 0.939 | 0.815 | 0.873 | 0.941 | 0.544 | 0.733 | 0.883 | 0.800 |
| AA, %E | 0.517 | 0.943 | 0.600 | 0.311 | 0.836 | 0.717 | 0.768 | 0.232 | 0.456 | 0.270 |
| EPA, %E | 0.278 | 0.473 | 0.976 | 0.357 | 0.716 | 0.110 | 0.134 | 0.157 | 0.959 | 0.351 |
| DHA, %E | 0.211 | 0.405 | 0.864 | 0.378 | 0.871 | 0.107 | 0.072 | 0.050 | 0.934 | 0.084 |

**Table S9.** *Cont.*

| **Dietary variable** | **Black** | | | | | **White** | | | | |
| --- | --- | --- | --- | --- | --- | --- | --- | --- | --- | --- |
|  | **TAG, mmol/L** | **T-C, mmol/L** | **HDL-C, mmol/L** | **LDL-C, mmol/L** | **T-C:HDL-C Ratio** | **TAG, mmol/L** | **T-C, mmol/L** | **HDL-C, mmol/L** | **LDL-C, mmol/L** | **T-C:HDL-C Ratio** |
|  | ***IL-6* IVS4 +869 A>G *n* = 130** | | | | | ***IL-6* IVS4 +869 A>G *n* = 121** | | | | |
| Fat, %E | 0.049 | 0.788 | 0.989 | 0.993 | 0.848 | 0.862 | 0.703 | 0.817 | 0.529 | 0.957 |
| SFA, %E | 0.073 | 0.481 | 0.526 | 0.205 | 0.262 | 0.335 | 0.601 | 0.705 | 0.555 | 0.464 |
| MUFA, %E | 0.301 | 0.658 | 0.630 | 0.343 | 0.436 | 0.509 | 0.936 | 0.444 | 0.989 | 0.505 |
| PUFA, %E | 0.059 | 0.504 | 0.593 | 0.459 | 0.321 | 0.152 | 0.252 | 0.872 | 0.323 | 0.508 |
| P:S ratio | 0.433 | 0.614 | 0.288 | 0.338 | 0.170 | 0.157 | 0.206 | 0.971 | 0.265 | 0.392 |
| *n*-3 PUFA, %E | 0.828 | 0.969 | 0.104 | 0.495 | 0.111 | 0.930 | 0.740 | 0.408 | 0.415 | 0.312 |
| *n*-6 PUFA, %E | 0.074 | 0.555 | 0.638 | 0.481 | 0.381 | 0.106 | 0.207 | 0.706 | 0.364 | 0.592 |
| *n*-6:*n*-3 PUFA ratio | 0.166 | 0.459 | 0.089 | 0.149 | 0.029 | 0.585 | 0.618 | 0.260 | 0.825 | 0.477 |
| ALA, %E | 0.481 | 0.922 | 0.114 | 0.435 | 0.115 | 0.796 | 0.576 | 0.794 | 0.764 | 0.881 |
| LA, %E | 0.073 | 0.558 | 0.640 | 0.483 | 0.384 | 0.107 | 0.203 | 0.699 | 0.360 | 0.595 |
| AA, %E | 0.627 | 0.642 | 0.216 | 0.921 | 0.406 | 0.492 | 0.539 | 0.230 | 0.704 | 0.488 |
| EPA, %E | 0.616 | 0.850 | 0.326 | 0.552 | 0.275 | 0.594 | 0.230 | 0.047 | 0.785 | 0.315 |
| DHA, %E | 0.513 | 0.991 | 0.398 | 0.746 | 0.419 | 0.491 | 0.136 | 0.023 | 0.512 | 0.290 |

**Table S10.** *p*-values for interaction between dietary fat intake (%E) and *IL-6* polymorphisms (genotype) on serum lipids in 266 white and black adequate reporter women, adjusted for age, fat mass, and ethnicity.

| **Dietary variable** | **TAG, mmol/L** | **T-C, mmol/L** | **HDL-C, mmol/L** | **LDL-C, mmol/L** | **T-C:HDL-C Ratio** |
| --- | --- | --- | --- | --- | --- |
|  | ***IL-6* IVS3 +281 G>T *n* = 257** | | | | |
| Fat, %E | 0.565 | 0.522 | 0.276 | 0.057 | 0.038 |
| SFA, %E | 0.526 | 0.190 | 0.322 | 0.263 | 0.583 |
| MUFA, %E | 0.268 | 0.596 | 0.328 | 0.327 | 0.100 |
| PUFA, %E | 0.271 | 0.158 | 0.286 | 0.021 | 0.133 |
| P:S ratio | 0.738 | 0.059 | 0.435 | 0.041 | 0.475 |
| *n*-3 PUFA, %E | 0.589 | 0.230 | 0.332 | 0.134 | 0.014 |
| *n*-6 PUFA, %E | 0.306 | 0.162 | 0.250 | 0.026 | 0.103 |
| *n*-6:*n*-3 PUFA ratio | 0.496 | 0.056 | 0.704 | 0.006 | 0.055 |
| ALA, %E | 0.518 | 0.386 | 0.278 | 0.111 | 0.027 |
| LA, %E | 0.296 | 0.155 | 0.244 | 0.026 | 0.102 |
| AA, %E | 0.906 | 0.855 | 0.285 | 0.988 | 0.735 |
| EPA, %E | 0.492 | 0.309 | 0.718 | 0.336 | 0.110 |
| DHA, %E | 0.329 | 0.251 | 0.902 | 0.202 | 0.129 |
| ***IL-6* IVS4 +869 A>G *n* = 251** | | | | | |
| Fat, %E | 0.527 | 0.212 | 0.242 | 0.037 | 0.013 |
| SFA, %E | 0.669 | 0.644 | 0.987 | 0.596 | 0.596 |
| MUFA, %E | 0.989 | 0.444 | 0.407 | 0.139 | 0.041 |
| PUFA, %E | 0.097 | 0.195 | 0.133 | 0.029 | 0.017 |
| P:S ratio | 0.219 | 0.360 | 0.278 | 0.132 | 0.089 |
| *n*-3 PUFA, %E | 0.953 | 0.706 | 0.733 | 0.881 | 0.722 |
| *n*-6 PUFA, %E | 0.078 | 0.214 | 0.119 | 0.039 | 0.017 |
| *n*-6:*n*-3 PUFA ratio | 0.311 | 0.278 | 0.473 | 0.076 | 0.148 |
| ALA, %E | 0.963 | 0.746 | 0.620 | 0.936 | 0.790 |
| LA, %E | 0.077 | 0.214 | 0.114 | 0.039 | 0.017 |
| AA, %E | 0.477 | 0.998 | 0.284 | 0.733 | 0.238 |
| EPA, %E | 0.837 | 0.883 | 0.763 | 0.726 | 0.474 |
| DHA, %E | 0.865 | 0.865 | 0.742 | 0.883 | 0.814 |

**Table S11.** *p*-values for interaction between dietary fat intake (%E) and *IL-6* polymorphisms (allelic) on serum lipids in 266 white and black adequate reporter women, adjusted for age, fat mass and ethnicity.

| **Dietary variable** | **TAG, mmol/L** | **T-C, mmol/L** | **HDL-C, mmol/L** | **LDL-C, mmol/L** | **T-C:HDL-C Ratio** |
| --- | --- | --- | --- | --- | --- |
|  | ***IL-6* IVS3 +281 G>T *n* = 257** | | | | |
| Fat, %E | 0.998 | 0.939 | 0.444 | 0.698 | 0.245 |
| SFA, %E | 0.973 | 0.086 | 0.490 | 0.108 | 0.584 |
| MUFA, %E | 0.229 | 0.326 | 0.370 | 0.208 | 0.087 |
| PUFA, %E | 0.474 | 0.203 | 0.291 | 0.391 | 0.622 |
| P:S ratio | 0.763 | 0.095 | 0.175 | 0.215 | 0.700 |
| *n*-3 PUFA, %E | 0.290 | 0.491 | 0.106 | 0.157 | 0.004 |
| *n*-6 PUFA, %E | 0.456 | 0.208 | 0.228 | 0.451 | 0.505 |
| *n*-6:*n*-3 PUFA ratio | 0.357 | 0.169 | 0.901 | 0.129 | 0.155 |
| ALA, %E | 0.572 | 0.225 | 0.130 | 0.037 | 0.008 |
| LA, %E | 0.454 | 0.195 | 0.212 | 0.439 | 0.489 |
| AA, %E | 0.965 | 0.915 | 0.544 | 0.912 | 0.856 |
| EPA, %E | 0.175 | 0.582 | 0.406 | 0.437 | 0.037 |
| DHA, %E | 0.154 | 0.438 | 0.607 | 0.385 | 0.068 |
| ***IL-6* IVS4 +869 A>G *n* = 251** | | | | | |
| Fat, %E | 0.290 | 0.464 | 0.624 | 0.736 | 0.647 |
| SFA, %E | 0.593 | 0.548 | 0.840 | 0.457 | 0.280 |
| MUFA, %E | 0.825 | 0.962 | 0.377 | 0.615 | 0.222 |
| PUFA, %E | 0.023 | 0.211 | 0.614 | 0.500 | 0.799 |
| P:S ratio | 0.053 | 0.230 | 0.738 | 0.415 | 0.497 |
| *n*-3 PUFA, %E | 0.812 | 0.775 | 0.569 | 0.982 | 0.341 |
| *n*-6 PUFA, %E | 0.018 | 0.203 | 0.500 | 0.550 | 0.966 |
| *n*-6:*n*-3 PUFA ratio | 0.082 | 0.364 | 0.848 | 0.536 | 0.459 |
| ALA, %E | 0.608 | 0.736 | 0.300 | 0.903 | 0.293 |
| LA, %E | 0.018 | 0.204 | 0.477 | 0.565 | 0.998 |
| AA, %E | 0.239 | 0.981 | 0.423 | 0.906 | 0.497 |
| EPA, %E | 0.652 | 0.598 | 0.855 | 0.491 | 0.444 |
| DHA, %E | 0.683 | 0.552 | 0.517 | 0.578 | 0.759 |
| Fat, %E | 0.290 | 0.464 | 0.624 | 0.736 | 0.647 |

**Table S12.** *p*-values for 3-way interaction between ethnic group, dietary fat intake (%E) and *IL-6* polymorphisms (genotype) on serum lipids in 266 white and black adequate reporter women, adjusted for age and fat mass.

| **Dietary variable** | **TAG, mmol/L** | **T-C, mmol/L** | **HDL-C, mmol/L** | **LDL-C, mmol/L** | **T-C:HDL-C Ratio** |
| --- | --- | --- | --- | --- | --- |
|  | ***IL-6* IVS3 +281 G>T *n* = 257** | | | | |
| Fat, %E | 0.512 | 0.259 | 0.957 | 0.962 | 0.429 |
| SFA, %E | 0.424 | 0.241 | 0.856 | 0.605 | 0.872 |
| MUFA, %E | 0.815 | 0.418 | 0.980 | 0.536 | 0.504 |
| PUFA, %E | 0.725 | 0.077 | 0.975 | 0.566 | 0.228 |
| P:S ratio | 0.758 | 0.249 | 0.990 | 0.570 | 0.348 |
| *n*-3 PUFA, %E | 0.348 | 0.455 | 0.156 | 0.063 | 0.134 |
| *n*-6 PUFA, %E | 0.680 | 0.085 | 0.961 | 0.512 | 0.376 |
| *n*-6:*n*-3 PUFA ratio | 0.500 | 0.015 | 0.224 | 0.077 | 0.151 |
| ALA, %E | 0.604 | 0.306 | 0.141 | 0.022 | 0.052 |
| LA, %E | 0.679 | 0.089 | 0.963 | 0.518 | 0.387 |
| AA, %E | 0.045 | 0.054 | 0.692 | 0.668 | 0.576 |
| EPA, %E | 0.087 | 0.639 | 0.515 | 0.445 | 0.484 |
| DHA, %E | 0.282 | 0.570 | 0.510 | 0.619 | 0.617 |
| ***IL-6* IVS4 +869 A>G *n* = 251** | | | | | |
| Fat, %E | 0.476 | 0.591 | 0.358 | 0.683 | 0.137 |
| SFA, %E | 0.352 | 0.725 | 0.583 | 0.946 | 0.374 |
| MUFA, %E | 0.488 | 0.404 | 0.157 | 0.534 | 0.105 |
| PUFA, %E | 0.889 | 0.887 | 0.605 | 0.523 | 0.389 |
| P:S ratio | 0.886 | 0.409 | 0.497 | 0.639 | 0.614 |
| *n*-3 PUFA, %E | 0.099 | 0.275 | 0.368 | 0.179 | 0.231 |
| *n*-6 PUFA, %E | 0.869 | 0.872 | 0.582 | 0.529 | 0.590 |
| *n*-6:*n*-3 PUFA ratio | 0.635 | 0.972 | 0.807 | 0.280 | 0.992 |
| ALA, %E | 0.046 | 0.093 | 0.223 | 0.007 | 0.011 |
| LA, %E | 0.856 | 0.885 | 0.571 | 0.520 | 0.609 |
| AA, %E | 0.167 | 0.080 | 0.494 | 0.963 | 0.601 |
| EPA, %E | 0.127 | 0.522 | 0.916 | 0.905 | 0.865 |
| DHA, %E | 0.414 | 0.568 | 0.909 | 0.862 | 0.819 |

**Table S13.** *p*-values for 3-way interaction between ethnic group, dietary fat intake (%E) and *IL-6* polymorphisms (allelic) on serum lipids in 266 white and black adequate reporter women, adjusted for age and fat mass.

| **Dietary variable** | **TAG, mmol/L** | **T-C, mmol/L** | **HDL-C, mmol/L** | **LDL-C, mmol/L** | **T-C:HDL-C Ratio** |
| --- | --- | --- | --- | --- | --- |
|  | ***IL-6* IVS3 +281 G>T *n* = 257** | | | | |
| Fat, %E | 0.298 | 0.496 | 0.816 | 0.767 | 0.967 |
| SFA, %E | 0.213 | 0.641 | 0.550 | 0.873 | 0.472 |
| MUFA, %E | 0.264 | 0.957 | 0.628 | 0.831 | 0.901 |
| PUFA, %E | 0.891 | 0.934 | 0.846 | 0.918 | 0.894 |
| P:S ratio | 0.961 | 0.533 | 0.395 | 0.782 | 0.555 |
| *n*-3 PUFA, %E | 0.598 | 0.502 | 0.961 | 0.586 | 0.690 |
| *n*-6 PUFA, %E | 0.969 | 0.835 | 0.767 | 0.883 | 0.965 |
| *n*-6:*n*-3 PUFA ratio | 0.938 | 0.773 | 0.845 | 0.805 | 0.982 |
| ALA, %E | 0.795 | 0.892 | 0.583 | 0.812 | 0.986 |
| LA, %E | 0.979 | 0.833 | 0.770 | 0.877 | 0.954 |
| AA, %E | 0.969 | 0.531 | 0.952 | 0.449 | 0.536 |
| EPA, %E | 0.419 | 0.283 | 0.360 | 0.534 | 0.771 |
| DHA, %E | 0.432 | 0.131 | 0.371 | 0.261 | 0.583 |
| ***IL-6* IVS4 +869 A>G *n* = 251** | | | | | |
| Fat, %E | 0.211 | 0.800 | 0.831 | 0.653 | 0.974 |
| SFA, %E | 0.072 | 0.848 | 0.773 | 0.430 | 0.499 |
| MUFA, %E | 0.171 | 0.762 | 0.728 | 0.612 | 0.976 |
| PUFA, %E | 0.675 | 0.486 | 0.452 | 0.774 | 0.915 |
| P:S ratio | 0.316 | 0.444 | 0.358 | 0.877 | 0.780 |
| *n*-3 PUFA, %E | 0.802 | 0.763 | 0.110 | 0.299 | 0.080 |
| *n*-6 PUFA, %E | 0.526 | 0.417 | 0.336 | 0.788 | 0.940 |
| *n*-6:*n*-3 PUFA ratio | 0.980 | 0.857 | 0.053 | 0.441 | 0.080 |
| ALA, %E | 0.674 | 0.623 | 0.509 | 0.301 | 0.106 |
| LA, %E | 0.535 | 0.414 | 0.330 | 0.784 | 0.942 |
| AA, %E | 0.760 | 0.957 | 0.691 | 0.999 | 0.947 |
| EPA, %E | 0.641 | 0.334 | 0.026 | 0.817 | 0.252 |
| DHA, %E | 0.408 | 0.196 | 0.013 | 0.556 | 0.283 |

© 2014 by the authors; licensee MDPI, Basel, Switzerland. This article is an open access article distributed under the terms and conditions of the Creative Commons Attribution license (http://creativecommons.org/licenses/by/3.0/).
